# Ultrafast and long-time excited state kinetics of an NIR-emissive vanadium(iii) complex II. Elucidating triplet-to-singlet excited-state dynamics†

**DOI:** 10.1039/d1sc02149d

**Published:** 2021-05-26

**Authors:** J. Patrick Zobel, Thomas Knoll, Leticia González

**Affiliations:** Institute of Theoretical Chemistry, Faculty of Chemistry, University of Vienna Währingerstr. 19 1090 Vienna Austria patrick.zobel@univie.ac.at leticia.gonzalez@univie.ac.at; Vienna Research Platform on Accelerating Photoreaction Discovery, University of Vienna Währingerstr. 19 1090 Vienna Austria

## Abstract

We report the non-adiabatic dynamics of V^III^Cl_3_(ddpd), a complex based on the Earth-abundant first-row transition metal vanadium with a d^2^ electronic configuration which is able to emit phosphorescence in solution in the near-infrared spectral region. Trajectory surface-hopping dynamics based on linear vibronic coupling potentials obtained with CASSCF provide molecular-level insights into the intersystem crossing from triplet to singlet metal-centered states. While the majority of the singlet population undergoes back-intersystem crossing to the triplet manifold, 1–2% remains stable during the 10 ps simulation time, enabling the phosphorescence described in Dorn *et al. Chem. Sci.*, 2021, DOI: 10.1039/D1SC02137K. Competing with intersystem crossing, two different relaxation channels *via* internal conversion through the triplet manifold occur. The nuclear motion that drives the dynamics through the different electronic states corresponds mainly to the increase of all metal–ligand bond distances as well as the decrease of the angles of trans-coordinated ligand atoms. Both motions lead to a decrease in the ligand-field splitting, which stabilizes the interconfigurational excited states populated during the dynamics. Analysis of the electronic character of the states reveals that increasing and stabilizing the singlet population, which in turn can result in enhanced phosphorescence, could be accomplished by further increasing the ligand-field strength.

## Abstract 2

The ultrafast triplet-to-singlet mechanism, responsible for the photoluminescence of the open-shell V^III^Cl_3_(ddpd) complex – based on Earth-abundant vanadium – is unraveled using non-adiabatic dynamics in full dimensionality.

## Graphical abstract

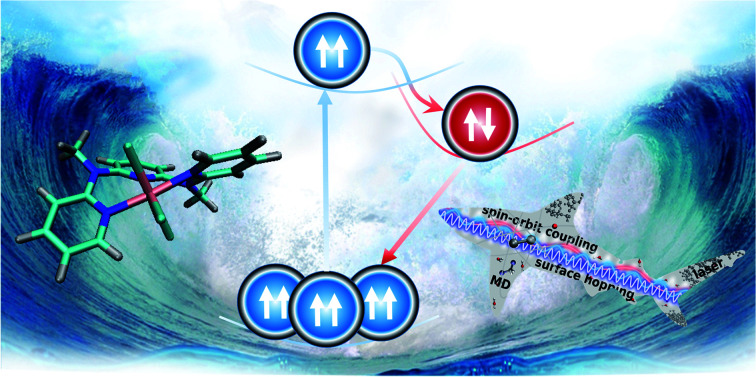

## 1 Introduction

The development of photoactive coordination compounds largely focuses on 4d or 5d metals because they often feature long-lived excited states that can be exploited in multiple applications. Such long-lived states are due to the large ligand-field splitting that increases the energy of metal-centered (MC) states, which are then inaccessible for relaxation.^1^ In contrast, complexes based on 3d metals have weaker ligand-field splitting and typically show rapid non-radiative relaxation through MC states.^2^ For practical applications, however, 4d/5d metals face the severe disadvantage that their rare natural occurrence makes them costly.^3^ Thus, significant effort is currently directed to the development of photoactive Earth-abundant 3d transition metal compounds, as low-cost alternatives, *e.g.* of photosensitizers.^4–6^

So far, 3d-metal complexes of chromium(0),^7^ iron(iii),^8,9^ cobalt(iii),^10^ and copper(i)^11^ exhibit luminescence in the visible region of the light spectrum. At longer wavelengths, luminescence has been observed for homoleptic chromium(iii) complexes using the ddpd,^12,13^ H_2_tpda^14^ and dqp^15^ ligands (ddpd = *N*,*N*′-dimethyl-*N*,*N*′-dipyridine-2-ylpyridine-2,6-diamine; H_2_tpda = 2,6-bis(2-pyridylamino)pyridine; dqp = 2,6-di(quinolin-8-yl)pyridine), which emit at the beginning of in the near-infrared region of the light spectrum (NIR) at 775, 782, and 747 nm, respectively. Using the ddpd ligand and vanadium(iii) in place of chromium(iii), the emission wavelength can be shifted to considerably longer wavelengths. Indeed, for the homoleptic [V^III^(ddpd)_2_]^3+^ complex, luminescence was found in the NIR-II range around 1100 nm,^16^ paving the way for promising applications in different fields, from photovoltaics to bio-imaging.^17,18^

**Fig. 1:**
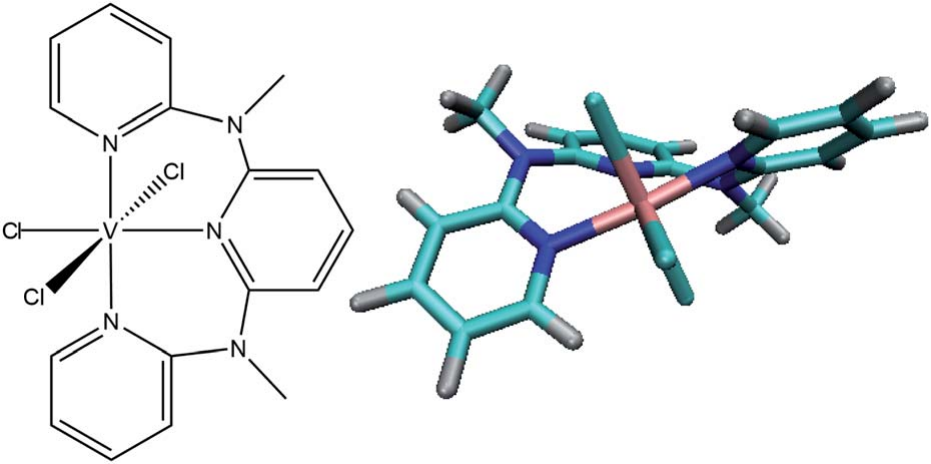
Structure of V^III^Cl_3_(ddpd) investigated in this work.

Here we present the heteroleptic chlorido-derivative V^III^Cl_3_(ddpd)^19^ (see Fig. 1), another exciting candidate for photoluminescence in the NIR-II range, which is able to emit at wavelengths of 1111 and 1219 nm at room temperature. While in paper I (see ref. 20) the experimental spectroscopic and electrochemical characterization of V^III^Cl_3_(ddpd) aided by static CASSCF-NEVPT2 calculations is presented, this work focuses on the electronic excited states and their time-dependent non-adiabatic evolution.

A mechanistic understanding of photoluminescence can help identifying the competing non-adiabatic relaxation pathways in a molecule. This can enable a better modulation of quantum yields, which ultimately leads to design principles for improving luminescence in complexes based on Earth-abundant transition metals. In this respect, non-adiabatic molecular dynamics simulations are indispensable because spectroscopic experiments cannot access details on the motion of the atoms of the system. However, simulations of coupled electronic and nuclear degrees of freedom in transition metal complexes are extremely demanding for several reasons. One is the size of the molecules, which prevents dynamical studies in full dimensionality including all nuclear degrees of freedom.^21–23^ Another is the large number of closely lying (near-degenerate) electronic states that can participate in the photodynamics and need to be taken into account. In order to overcome these challenges, we recently combined the non-adiabatic trajectory surface hopping method SHARC^24,25^ with a linear vibronic coupling (LVC) approach^26^ to compute the electronic potentials.^27^ In this way, the photodynamics of a number of transition metal complexes have been investigated,^16,28–30^ where time-dependent density functional theory (TDDFT) was used to parametrize the LVC potentials. Given its positive trade-off between accuracy and computational cost, TDDFT is likely the most popular method to study transition metal complexes,^31,32^ and it has also been applied in on-the-fly surface hopping studies of other transition-metal complexes.^33–37^ However, TDDFT can become problematic for open-shell systems with degenerate ground states due to its single-reference nature. This is the case for pseudo-octahedral vanadium(iii) complexes, such as V^III^Cl_3_(ddpd), which possesses a d^2^ ground-state electron configuration.

Here we present full-dimensional non-adiabatic photodynamics simulations of V^III^Cl_3_(ddpd) using trajectory surface hopping molecular dynamics in solution based on complete-active space self-consistent field (CASSCF)-derived LVC potentials. We show that CASSCF is able to describe all low-lying MC states expected from ligand-field theory. The calculation of the entire set of MC states is only possible with a multi-configurational method, as demonstrated by a transition-density matrix analysis. This analysis also reveals the presence of an important higher-order excitation character in low-lying excited states with ligand contributions, which are missing in methods relying on single excitations such as TDDFT. This work emphasizes the need for going beyond single-reference methods to obtain even a qualitatively correct electronic structure in V^III^Cl_3_(ddpd). The demanding CASSCF-based nonadiabatic dynamics simulations uncover the population pathways from the triplet to the singlet excited MC states *via* intersystem crossing (ISC), providing new insight into the luminescent states of V^III^Cl_3_(ddpd) and strategies to enhance the luminescence properties of related 3d metal complexes.

## 2 Methods

### 2.1 Trajectory surface hopping simulations

The nonadiabatic dynamics of V^III^Cl_3_(ddpd) including all 123 normal modes were simulated using an LVC model within the SHARC surface hopping approach.^27,38^ Trajectories were set up using 2000 initial coordinates and momenta from a Wigner distribution of the ground-state PES.^39,40^ The initial excited states were selected stochastically within an energy range of 0.5 eV around the maximum of the second absorption band (3.0–3.5 eV) based on their oscillator strength.^41^ Accordingly, 2000 trajectories were started in the T_6_ (664), T_7_ (891), T_8_ (346), T_9_ (55), T_10_ (24), T_11_ (19), and T_12_ (1). The trajectories were then propagated for 10 ps using a nuclear time step of 0.5 fs and an electronic time step of 0.02 fs within the local diabatization method.^42^ In total, this amounts to 20 ns of non-adiabatic dynamics carried out with the PySHARC driver,^27^ which allows for efficient in-memory communication between a Python interface and the routines of SHARC. An energy-based decoherence correction with a constant of *C* = 0.1 a.u. was used.^43^ During the surface hops, the kinetic energy was adjusted by re-scaling the velocity vectors. Surface hopping probabilities were approximated using the wave function overlaps described in ref. 44.

### 2.2 Linear vibronic coupling model

In an LVC model, the potential energy surfaces (PESs) are approximated in terms of the ground-state PES *V*_0_ and the first-order (linear) vibronic coupling term ***W*** on the basis of the mass-frequency scaled normal mode coordinate ***Q***

1 ***V***(***Q***) = *V*_0_(***Q***)**1** + ***W***(***Q***)

The ground-state PESs are approximated as harmonic oscillators with frequencies *ω*_*i*_, *i.e.*,2
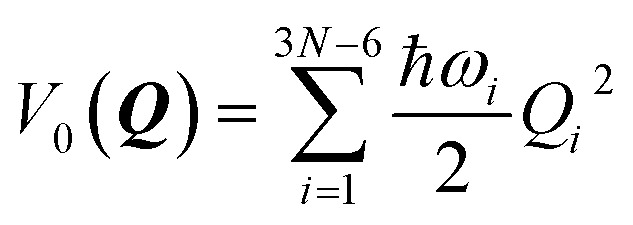


The coupling term reads3
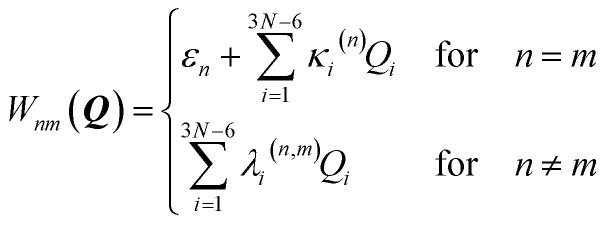
where *ε*_*n*_ are the vertical excitation energies at the Franck–Condon (FC) geometry, while *κ*_*i*_^(*n*)^ and *λ*_*i*_^(*n*,*m*)^ are the intrastate and interstate coupling elements for the normal mode coordinate *Q*_*i*_. The coupling elements were obtained numerically from CASSCF calculations (see below) on geometries displaced by ±0.05 units from the ground-state equilibrium geometry for each normal mode. The intrastate coupling *κ*_*i*_^(*n*)^ values were obtained as numerical gradients and the interstate couplings *λ*_*i*_^(*n*,*m*)^ were obtained from the change in the wavefunction overlaps. The spin–orbit couplings (SOCs) in the LVC model potential were approximated by constant SOCs obtained at the ground-state equilibrium geometry.

The absorption spectrum and the density-of-states were calculated from a Wigner distribution of 20 000 geometries using the LVC model potential.

### 2.3 Electronic structure calculations

The triplet ground state of V^III^Cl_3_(ddpd) in acetonitrile (MeCN) was optimized using unrestricted B3LYP,^45,46^ the def2-TZVP basis set,^47^ and the D3 dispersion correction using Becke–Johnson damping (D3BJ)^48^ as implemented in the ORCA4.1 program package.^49^ A numerical frequency calculation at the same level of theory confirms that no imaginary frequencies are obtained. Solvent effects were modeled using the conductor polarizable continuum model (C-PCM). For the self-consistent-field (SCF) calculations, the resolution-of-identity approximation (RIJCOSX), tight SCF convergence criteria (TightSCF), and the Grid4 integration grid were used. For the geometry optimization, tight convergence criteria (TightOpt) were used. Scalar relativistic effects are taken into account with the zeroth-order regular approximation^50^ (ZORA). Cartesian coordinates are given in Table S1 in the ESI.†

The electronic excited states of V^III^Cl_3_(ddpd) in MeCN were calculated with CASSCF^51^ and the ANO-RCC-VDZP basis set^52,53^ using the v18.0.o180122-0800 version of the OpenMolcas program package.^54^ Thereby, 16 triplet and 15 singlet states were calculated at the state-averaged CASSCF level of theory with equal weights. An active space comprising 10 electrons in 13 orbitals was used (10,13). These orbitals correspond mainly to the five metal d orbitals, four ligand π orbitals, and four ligand π* orbitals; they are shown in Fig. S1 in the ESI.† A closer analysis also shows contributions from the p orbitals of the chlorido ligands in the active orbitals, which result in a small participation of the chlorido ligands in the charge flow during the excitation. Solvent effects of MeCN were described using the C-PCM model. SOCs between all states were calculated using the atomic mean field (AMFI) approximation^55^ in the restricted active space state interaction (RASSI) program in OpenMolcas.

In addition, to assess the performance of the CASSCF results, the energies of the 16 triplet and 15 singlet states were also calculated with the multi-state complete active space second-perturbation theory (MS-CASPT2)^56^ using an imaginary level shift of 0.1 a.u. (ref. 57) and IPEA shift values^58,59^ of 0.0 a.u. and 0.25 a.u. The corresponding results are discussed in Section S3 in the ESI.†

### 2.4 Quantitative wavefunction analysis

Electronically excited states are often described in terms of the canonical orbitals that characterize the most important configurations in the excited-state wave functions.^60^ In the presence of many configurations, this description becomes tedious and often misleading. A more comprehensible and quantitative analysis of the wavefunctions can be obtained by analyzing the one-particle transition-density matrix.^61^ For electronic structure methods including only single excitations, the one-particle transition-density matrix contains the full information of the excited-state wave functions. For electronic structure methods including higher excitation levels, such as CASSCF, this description is only approximate. A singular-value decomposition of the transition-density matrix provides a set of hole and electron natural transition orbitals that give a compact representation of the excited-state wave function – particularly in cases where a single pair of hole and electron natural-transition orbitals represents ∼99% of the character of the excitation. This orbital representation is convenient when analyzing the electronic structure at a single geometry. For multiple geometries, it is not possible to compute meaningful averaged orbitals in a straightforward manner. In these cases, it is possible to partition the molecule into several fragments and compute the contributions of the hole and electron parts of the fragments to the full transition density matrix. The fragments can comprise several atoms, *e.g.*, a ligand, or correspond to individual atoms; importantly, their representation is invariant to the displacement of the nuclei. This procedure provides an easily understandable illustration of the charge flow that occurs during the dynamics in the different electronic states. The analysis of the transition-density matrix has been performed using the TheoDORE routine^62^ integrated in the WFA module in OpenMolcas.^54^

## 3 Results and discussion

### 3.1 Electronic structure calculations

**Fig. 2:**
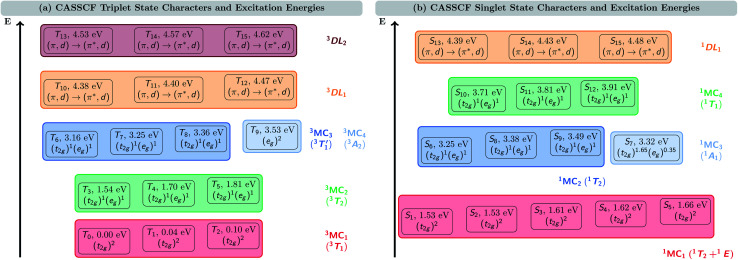
Characters and energies of the (a) triplet and (b) singlet states calculated with CASSCF(10,13). Note that the energy axis is not on scale. ^3,1^DL denotes d-assisted ligand excited states, while other state labels follow ligand-field terms of octahedral complexes.

Fig. 2 shows the triplet (a) and singlet (b) electronic states of V^III^Cl_3_(ddpd) calculated at the CASSCF(10,13) level of theory. As the vanadium metal center possesses a d^2^ ground-state electron configuration, the ground state of V^III^Cl_3_(ddpd) is a triplet state, T_0_. Only slightly above T_0_, we find two further triplet states, T_1_ at 0.04 eV (332 cm^−1^) and T_2_ 0.10 eV (799 cm^−1^). Since in V^III^Cl_3_(ddpd) the vanadium center has an almost octahedral coordination sphere, the three states T_0_–T_2_ correspond to the three components of the ^3^T_1_ term – the ground state term predicted by ligand-field theory (see the Tanabe–Sugano diagram in Fig. S2 in the ESI†). The CASSCF(10,13) natural orbital occupation numbers (Table S4 in the ESI†) show that in all three states the d electrons occupy two of the d_*xy*_, d_*yz*_, and d_*xz*_ orbitals, and so they can be assigned the (t_2g_)^2^ configuration. To avoid confusion between the T_n_ nomenclature of triplet states ordered by energy and the ^3^T_n_ term symbol, here we also label these three states as ^3^MC_1_ states. Thereby, MC refers to the metal-centered character of these states, and we implicitly retain the threefold spatial degeneracy of the ^3^T_1_ term.

The higher-lying triplet states are described likewise. Between 1.54 and 1.81 eV (12 460–14 577 cm^−1^), we find the three components of the ^3^T_2_ term of the (t_2g_)^1^(e_g_)^1^ configuration, that will be referred to as (threefold nearly-degenerate) ^3^MC_2_ states. At energies of 3.16–3.53 eV (25 505–28 507 cm^−1^), there are four nearly degenerate states. Three of these states (T_6_–T_8_) also possess the (t_2g_)^1^(e_g_)^1^ configuration: these states can be assigned to the 
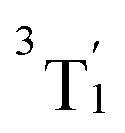
 term (threefold nearly-degenerate ^3^MC_3_ states). The fourth state, T_9_, possesses a (e_g_)^2^ configuration; it is assigned to the ^3^A_2_ term (non-degenerate ^3^MC_4_ state). Finally, we find two sets of three states each, T_10_–T_12_ between 4.38 and 4.47 eV (35 303–36 046 cm^−1^)) and T_13_–T_15_ between 4.53 and 4.62 eV (36 546–37 285 cm^−1^), respectively. Based on the natural orbital occupation numbers (Table S4 in the ESI†), these states correspond to the transition of an electron from a ddpd π orbital to a ddpd π* orbital. However, these states are actually a linear combination of equal parts of π → π* single excitations and (π, d) → (π*, d) double excitations, as becomes apparent when analyzing the one-particle transition density (see discussion below). Thus, we label these sets of states as the d-assisted ligand-centered states ^3^DL_1_ and ^3^DL_2_, respectively.

The S_1_–S_15_ singlet states are shown in Fig. 2(b). As can be seen, there is a group of five states (S_1_–S_5_) at energies between 1.53 and 1.66 eV (12 329–13 362 cm^−1^). These states all share a (t_2g_)^2^ electron configuration. They correspond to the three components of the ^1^T_2_ term and the two components of the ^1^E term, which are predicted as the lowest-energy singlet states in the Tanabe–Sugano diagram (Fig. S2 in the ESI†), where they are almost degenerate. In this case, it is harder to distinguish which of the states S_1_–S_5_ corresponds to each of the two terms due to the small energy gap (0.13 eV, 1033 cm^−1^) as well as their shared electron configuration. Therefore, and for the sake of simplicity, we denote these five states only as ^1^MC_1_ states.

At higher energy, a group of four states appears between 3.25 and 3.49 eV (26 221–28 167 cm^−1^). Three of them (S_6_, S_8_, and S_9_) share the (t_2g_)^1^(e_g_)^1^ electron configuration. The remaining S_7_ state is described by a (t_2g_)^1.65^(e_g_)^0.35^ configuration. For this state, the non-integer occupation numbers of the natural orbitals originate from the multi-configurational nature of the CASSCF wave function. We note that the natural orbital occupation numbers for all other electronic states lie much closer to 0, 1, or 2, *i.e.*, usually differing by less than 0.1 from these integer values. Following their different electronic configurations, we ascribe the S_6_, S_8_, and S_9_ states to the ^1^T_2_ term (threefold nearly-degenerate ^1^MC_2_ states), while S_7_ is assigned to the ^1^A_1_ term (non-degenerate ^1^MC_3_ state).

Between 3.71 and 3.91 eV (29 913–31 509 cm^−1^), we find a further group of three singlet states that share the (t_2g_)^1^(e_g_)^1^ configuration, which we assign to the ^1^T_1_ term (threefold nearly-degenerate ^1^MC_4_ states). Finally, we reach a group of three singlet states between 4.39 and 4.48 eV (35 429–36 149 cm^−1^) with similar features as the ^3^DL_1,2_ triplet states. Based on the natural orbital occupation numbers, these states correspond to the π → π* excitation; however, a closer analysis reveals additional (π, d) → (π*, d) double excitation character, which will be discussed in more detail below.

The analysis of the CASSCF natural orbitals provides a detailed, yet only qualitative description of the electronic states of V^III^Cl_3_(ddpd). One strategy to obtain a quantitative description is to analyze the transition density matrix (TDM) *γ*_*IJ*_, which describes the change in electron density associated with a transition from the initial state *Ψ*_*I*_—here the ground state *Ψ*_0_—to the final state *Ψ*_*J*_. This can be easily done for electronic structure methods including only single excitations, such as TDDFT, as in this case one needs to compute only the one-particle TDM *γ*_*IJ*_^(1)^. For methods like CASSCF that include higher-order excitations, one should also analyze higher-order TDMs *γ*_*IJ*_^(*n*)^. Unfortunately, there is no computer program available yet that offers this possibility. Thus, here we are content with scrutinizing the one-particle TDM *γ*_*IJ*_^(1)^ of the CASSCF electronic states to infer the limitations of the single-excitation character in the different electronic states. For simplicity, henceforth, we drop the superscript and refer to the one-particle TDM just by *γ*_*IJ*_.

**Fig. 3:**
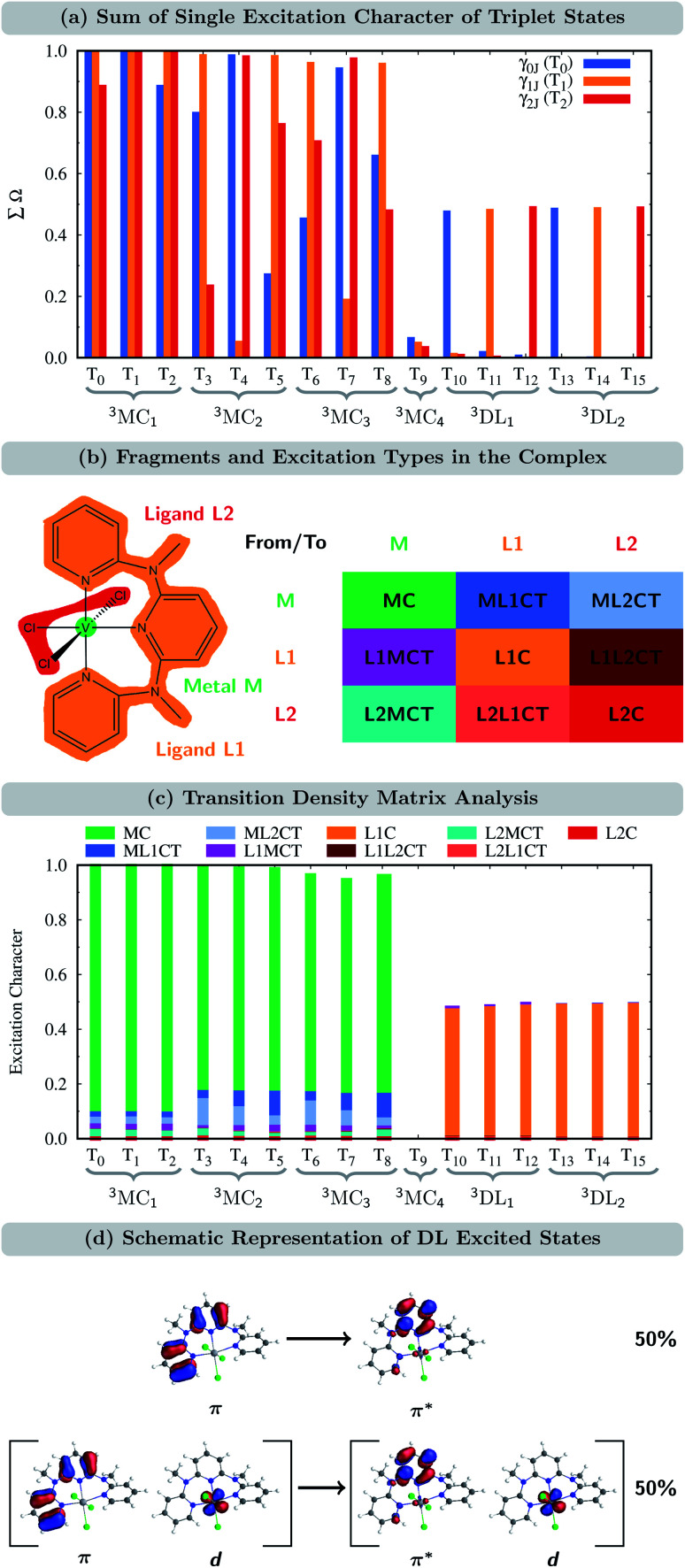
(a) Sum of single excitation character *∑Ω* for the triplet states calculated from the transition density matrices *γ*_0*J*_, *γ*_1*J*_, and *γ*_2*J*_ of the T_0_, T_1_, and T_2_ states, respectively. Note that *∑Ω* is set to 1 for *γ*_*II*_ to indicate that a state can be described quantitatively using itself as a reference when taking into account only single excitations. (b) Fragment partition and classification of excited-state characters in V^III^Cl_3_(ddpd). (c) Transition density matrix analysis of the triplet states. Analysis used the transition-density matrix with the largest *∑Ω* out of *γ*_0*J*_, *γ*_1*J*_, and *γ*_2*J*_. (d) Characterization of DL excited states.

In order to verify to which extent the analysis of the one-particle TDM is appropriate for the CASSCF electronic states, we first determined the sum of the single excitations *∑Ω* of the CASSCF wave functions with respect to the ground state of V^III^Cl_3_(ddpd), *i.e.*, the T_0_ state, which is the lowest-energy component of the nearly-degenerate ^3^MC_1_ states. This quantity, *∑Ω* for *γ*_0*J*_, for all the triplets is shown in Fig. 3(a) in blue. Remarkably, only for the states T_1_, T_4_, and T_7_, the sum of single excitations *∑Ω* is close to 1. For two additional states (T_2_ and T_3_), *∑Ω* still lies between 80 and 90%; however, for the remaining triplet states, *∑Ω* is significantly smaller than one. Clearly, only a small subset of states can be accurately described as single excitations from the T_0_ reference state.

We recall that based on the natural orbital occupation numbers, the ground state of V^III^Cl_3_(ddpd) adopts the (t_2g_)^2^ configuration, while a number of excited states (^3^MC_2,3_) possess the (t_2g_)^1^(e_g_)^1^ configuration. At first glance, the latter states seem to be the result of promoting a single electron from a t_2g_ orbital to an e_g_ orbital. However, as revealed by the one-particle TDM analysis, their wave functions must also include higher-order excitations, leading to *∑Ω* values significantly smaller than one. Yet, it is possible to describe the (t_2g_)^1^(e_g_)^1^ excited states mainly by single excitations if a different reference state other than the T_0_ state is chosen. In particular, this can be achieved by selecting the T_1_ and T_2_ states, which are the complementary components of the triply nearly-degenerate ^3^MC_1_ term. This is shown by the orange and red bars in Fig. 3(a) that denote the TDM *γ*_1*J*_ and *γ*_2*J*_, which use the T_1_ and T_2_ states as a reference, respectively. Taking together all three one-particle TDMs *γ*_*IJ*_ (*I* = 0, 1, and 2), we now see that each of the states T_0_–T_8_ can be described by single excitations with *∑Ω* ≈ 1 for at least one of the reference states (at least one of the three bars is close to 1). Thus, each of the states T_0_–T_8_ is reasonably described by the one-particle TDM, if the appropriate reference state is selected. For the T_9_ state, we see in Fig. 3(a) that *∑Ω* for all three *γ*_*IJ*_ is close to zero. This is because the T_9_ state (^3^MC_4_) corresponds to the (e_g_)^2^ configuration, which naturally cannot be described by single excitations from (t_2g_)^2^.

For the states T_10_–T_15_ (^3^DL_1,2_), *∑Ω* amounts only to *ca.* 0.5 when using the appropriate reference state. Based on the natural orbital occupation numbers, these states feature the (t_2g_)^2^ configuration at the vanadium center and the π → π* excitation at the ddpd ligand. However, these states do not correspond to the simple π → π* excitation as, otherwise, *∑Ω* would amount to 1 for one of the TDMs. In order to clarify the character of the states T_10_–T_15_, we use charge transfer numbers^63^ and partition V^III^Cl_3_(ddpd) into three fragments (Fig. 3(b)): the metal center M, the ddpd ligand (ligand L1), and the three chlorido ligands (ligand L2). The CASSCF configurations can then be assigned to one of the nine different types of excitations sketched in Fig. 3(b): local excitations – either centered at the metal atom (MC) or at one of the two ligands (L1C, L2C) – metal-to-ligand charge transfer excitations (ML1CT, ML2CT), ligand-to-metal charge-transfer excitations (L1MCT, L2MCT), and ligand-to-ligand excitations (L1L2CT, L2L1CT). Fig. 3(c) collects the resulting charge-transfer numbers for each electronic state, see also Tables S6–S8 in the ESI.† As can be seen, the majority of the single excitation character of the T_10_–T_15_ states corresponds to L1C excitations, *i.e.*, excitations centered at the ddpd ligand. Combining this observation with the natural orbital occupation numbers leads to the conclusion that these states correspond to linear combinations of 50% π → π* single excitations and 50% (π, d) → (π*, d) double excitations, see Fig. 3(d). Accordingly, we label these states d-assisted ligand-centered (DL) states.

From the remaining states in Fig. 3(c) (the T_9_ state is excluded as it corresponds to a double excitation), one can see that the states T_0_–T_8_ are MC excitations, with small admixtures of ML1CT and ML2CT characters. In passing, we note that the admixtures of chloride orbitals with the vanadium d orbitals can only be observed in the orbitals using an iso value lower than 0.05 (*cf.* Fig. S1(a and b) in the ESI†). This further highlights the benefits of using the quantitative TDM analysis compared to the qualitative orbital-based description.

In the accompanying paper I,^20^ the higher-energy part of the absorption spectrum of V^III^Cl_3_(ddpd) has been calculated using LR-TDDFT. The TDDFT calculations predicted excited states with L1MCT and L1C characters following the manifold of MC states. Based on the double excitation character found for the ^3^DL states, the excitation energies of the L1C states are likely to be overestimated by LR-TDDFT – as LR-TDDFT includes only single excitations – while standard hybrid functionals such as the employed B3LYP functional also tend to underestimate the energies of CT states. It is also interesting to discuss whether other DFT-based methods such as spin-flip TDDFT^64^ (SF-DFT) or a ΔSCF approach^65^ should, in principle, be able to provide a better description of the electronic states of V^III^Cl_3_(ddpd) than “standard” LR-TDDFT. While SF-DFT can be used to describe multi-configurational ground states of open-shell systems^64^ by applying a LR-TDDFT approach from a spin-flipped reference state, this is only reasonable if the reference state itself can be described as a single configuration. As shown in Fig. 2, this is problematic due to the near-degeneracy of the excited states, both among the triplet and the singlet manifold. Even if it was possible to choose one of the two excited states with rather unique electronic configurations, *i.e.*, the S_7_ state or the T_9_ state, the subsequent success in SF-DFT calculations would be questionable. Due to the (e_g_)^2^ configuration of the T_9_ state, SF-DFT in a linear-response framework would not be capable of describing any of the ground-state components T_0_, T_1_, and T_2_. Furthermore, the (t_2g_)^1.65^(e_g_)^0.35^ configuration of the S_7_ state highlights its multi-reference character, making it inaccessible to single-reference methods. In ΔSCF methods, it is possible to compute single components of spatially degenerate excited states of different symmetry.^65^ However, as soon as this degeneracy is lifted, *e.g.*, by moving away from a highly-symmetric reference geometry, these states become inaccessible as well. Thus, we conclude that the correct description of the electronic states of V^III^Cl_3_(ddpd) requires multi-configurational methods including higher-order excitations.

### 3.2 Excited-state dynamics simulations

**Fig. 4:**
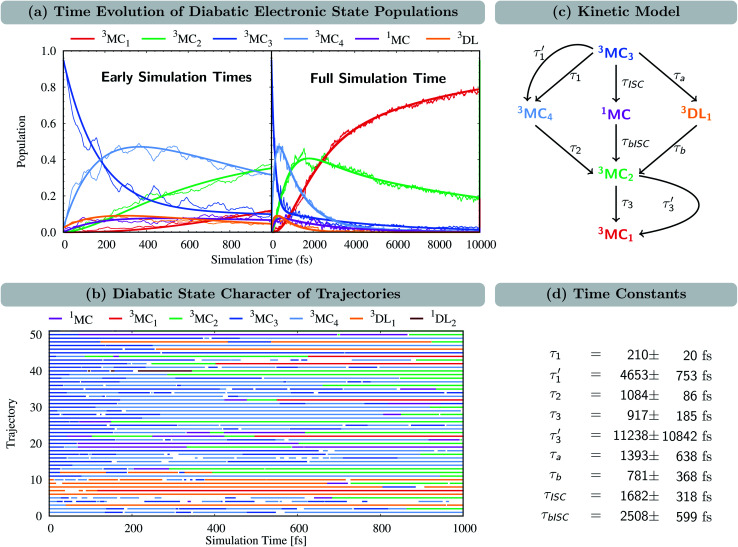
(a) Time-evolution of the diabatic electronic state populations. Thin lines represent state populations and thick lines represent fitted curves. (b) Time-resolved diabatic state character of 50 sample trajectories. Colors indicate the dominant character, summing up to at least 60% of the total population for each trajectory (this threshold allowed an average assignment of 95%, *i.e.* 19 000 of the 20 000 time steps for each trajectory; see Fig. S7† for a more strict threshold of 70%, which accounts for 87% of the simulation time). Blank spots indicate that no dominant state could be assigned at a particular time step. (c) Kinetic model used to fit the time evolution of the populations. (d) Fitted time constants including error estimates obtained using the bootstrap method with 100 copies.^66^

The time evolution of the *diabatic* electronic states, *i.e.*, states that are characterized by the same electronic configuration, is shown in Fig. 4(a). Since in the LVC model no intruder states can enter the dynamics simulation at any geometry, all electronic states can be described on the basis of the diabatic states of the equilibrium geometry at all simulation times. For the following analysis, we combine the population of the different components of the nearly degenerate ^3^MC_1_, ^3^MC_2_, and ^3^MC_3_ states into one population each. In addition, the populations of all singlet states were combined into one (^1^MC); individual diabatic singlet state populations can be found in Fig. S5 in the ESI.† For completeness, we also show the time evolution of the *adiabatic* states in Fig. S4 in the ESI.†

At *t* = 0 fs, the ^3^MC_3_ state is populated by 95%, while the remaining population is in the ^3^MC_4_ and ^3^DL_1_ states (2.5% each). Within the first 1 ps, the population of the ^3^MC_3_ state decreases to *ca.* 35%. Concomitantly, the population in the ^3^MC_4_ state increases up to *ca.* 45% until 300 fs and then decreases again. The population of ^3^MC_2_ also increases – albeit at a slower rate – reaching *ca.* 40% after 1 ps. Additionally, the ^3^MC_1_ and ^3^DL_1_ states are also populated in small amounts. Most importantly, the population is also transferred to the singlet states (^1^MC), reaching *ca.* 10% after 100 fs and remaining constant until 1 ps. At later times, all states depopulate strongly except ^3^MC_1_, illustrating how important it is to simulate dynamics beyond 1 ps. After 10 ps, the population of ^3^MC_1_ has reached *ca.* 80%, and the remaining *ca.* 20% is mainly in the ^3^MC_2_ state. The population of the singlet ^1^MC states decreases from its peak of 10% to values of 1–2% after 4 ps. Noticeably, the singlet population is almost entirely in the lowest singlet state ^1^MC_1_, as shown in Fig. S5 in the ESI.†

The time-evolution of the diabatic populations of 50 random trajectories is shown in Fig. 4(b) up to 1 ps (see also Fig. S6 in the ESI† for 10 ps). This allows us to easily follow the individual pathways of the trajectories through the different electronic states and propose a kinetic model to explain their time evolution. We see that trajectories in the ^3^MC_3_ state (dark blue) tend to change to either the ^3^MC_4_ state (light blue), the ^3^DL_1_ state (orange), or the singlet ^1^MC state (purple). From either of these states, trajectories then usually transfer to the ^3^MC_2_ state (green), from where they undergo internal conversion (IC) to the ^3^MC_1_ ground state (red). The ^3^DL_2_ states (brown) are rarely visited and thus henceforth disregarded. Based on these observations, we propose the following simplified kinetic model,4
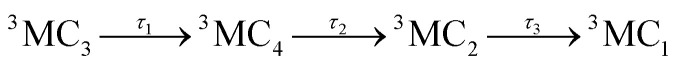
5
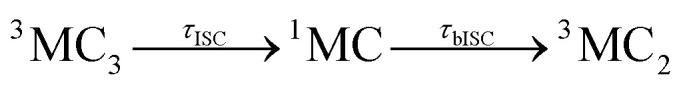
6
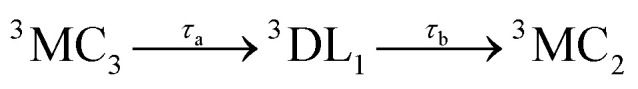
for which, fits for the populations are shown in Fig. S8 in the ESI.† As some of the fitted curves did not follow closely the state populations, we described the ^3^MC_3_ → ^3^MC_4_ and ^3^MC_2_ → ^3^MC_1_ reactions by a biexponential function, adding7
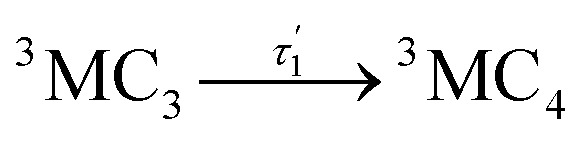
8
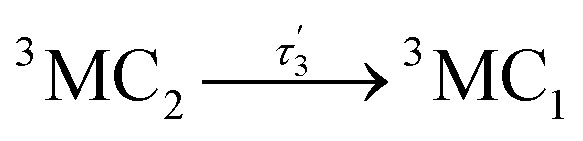
which improves the fit considerably: now all fit curves (thick lines in Fig. 4(a)) closely follow the population curves (thin lines). The complete kinetic model is shown in Fig. 4(c) and the fitted time constants are listed in Fig. 4(d).

Accordingly, IC *via*^3^MC_3_ → ^3^MC_4_ occurs with a fast time constant of *τ*_1_ = 210 ± 20 fs and a slow time constant of 
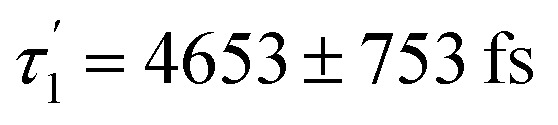
. Competing with that is both, ISC to the ^1^MC singlet states with a single time constant of *τ*_ISC_ = 1682 ± 318 fs and IC *via* the ^3^DL_1_ states with a time constant of *τ*_a_ = 1393 ± 638 fs. The time constant of ISC is thereby similar to the time constant *τ* = 1.5 ps that describes the rise of a long-lived signal in the transient absorption experiments in the accompanying paper I,^20^*i.e.*, which describes the population of the singlet states *via* ISC. Based on the time constants fitted in the simulations, we can calculate the yields of the three deactivation pathways that start from the ^3^MC_3_ state. In total, 79.2% of the excited-state population decays *via*^3^MC_3_ → ^3^MC_4_, 11.4% of the excited population decays *via*^3^MC_3_ → ^3^DL_1_, and 9.4% of the excited state population undergoes ISC to the ^1^MC states.

From these results, it follows how one can increase ISC to the ^1^MC states and stabilize the population in the singlet manifold, thus enhancing phosphorescence in compounds related to V^III^Cl_3_(ddpd). As discussed above, the main competing reaction to ^3^MC_3_ → ^1^MC ISC, that populates the singlet states, is IC *via*^3^MC_3_ → ^3^MC_4_. The ^3^MC_3_ and ^3^MC_4_ states are characterized by (t_2g_)^1^(e_g_)^1^ and (e_g_)^2^ configurations, respectively, while the majority of the singlet population is in the ^1^MC_1_ states described by the (t_2g_)^2^ configuration. The t_2g_ orbitals are non-bonding in ligand-field theory while the e_g_ orbitals possess an antibonding character. If the ligand-field strength was increased, it would lead to a destabilization of the e_g_ orbitals, which, in turn, would increase the energy of all electronic states where the e_g_ orbital is occupied. Thus, increasing the ligand-field strength would increase the energy of the ^3^MC_3_ and ^3^MC_4_ states while leaving the ^1^MC_1_ states unaffected. Furthermore, the increase would be more pronounced for the ^3^MC_4_ state than for the ^3^MC_3_ state, since in the ^3^MC_4_ state, the e_g_ orbitals are occupied twice. This could lead to a situation where ^3^MC_4_ is inaccessible during the dynamics, thus quenching the main competing pathway to ISC. Additionally, increasing the ligand-field strength can also stabilize the population in the singlet manifold, since the depopulation of the singlet states occurs *via*^1^MC → ^3^MC_2_. ^3^MC_2_ is characterized by the (t_2g_)^1^(e_g_)^1^ configuration. Thus, it will be shifted to higher energies compared to ^1^MC_1_ and may become inaccessible for depopulation of the singlet states as well.

**Fig. 5:**
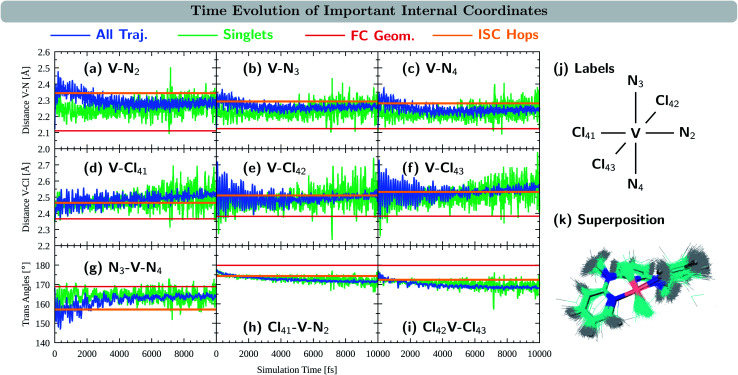
Time-evolution of important internal coordinates: (a–c) V–N distances, (d–f) V–Cl distances and (g–i) trans angles. Average values of the 10 000 trajectories in blue, the average of trajectories in singlet states in green, the value at the equilibrium geometry in red, and the average value at the ISC hopping geometries in orange. (j) Atom numbering. (k) Superposition of structures at the ISC hopping geometries.

We now turn to discuss the internal coordinates that show most distinct changes during the course of the simulations. These coordinates were identified by analysing the average motion of all trajectories on the basis of the normal modes of the reference equilibrium geometry,^67,68^ see also Section S4.6 in the ESI.† The identified coordinates include the distances V–N and V–Cl between the central vanadium atom and the ligating N and Cl atoms, as well as the angles X–V–Y between the trans-coordinated ligand atoms (X, Y = N, Cl). The average values of these internal coordinates from the 2000 trajectories are shown in Fig. 5(a–i) in blue. For comparison, the respective value at the equilibrium geometry is shown as a red line. Atom numbering is shown in Fig. 5(j). Note that the initial average values at *t* = 0 fs do resemble their reference values at the equilibrium geometry, as demonstrated by 0–200 fs zoom in Fig. S12 in the ESI.†

As seen in Fig. 5(a–c), the distances between the vanadium atom and the ligating N atoms in the ddpd moiety increase rapidly at the beginning of the dynamics from *ca.* 2.11–2.12 Å to 2.3–2.4 Å. The effect is most pronounced for the V–N_2_ distance – the vanadium and the nitrogen atom trans to a chlorido ligand – which reaches values close to 2.5 Å. At later times, all V–N distances decrease again, however, ending up at 2.31 Å (V–N_2_) and 2.25–2.27 Å (V–N_3,4_), *i.e.*, distances that are still considerably larger than in the equilibrium geometry. The evolution of the V–Cl distances, Fig. 5(d–f), also shows a rapid initial increase from values of 2.36–2.38 Å to the range of 2.4–2.6 Å. Thereby, the increase is smaller for the chlorido ligand that is coordinated trans to a ligating nitrogen atom (V–Cl_41_) than for the other two chlorido ligands. However, in contrast to the V–N distances, the V–Cl distances increase in average with longer simulation times, reaching 2.49–2.52 Å after 10 ps. The X–V–Y angles between the trans-coordinating atoms in V^III^Cl_3_(ddpd) are shown in Fig. 5(g–i). The N_3_–V–N_4_ angle decreases in the beginning of the dynamics from 169° to 150–160°, while at longer simulation times it increases again to values above 160° – however, still below the ground-state reference value. For the Cl_41_–V–N_2_ and Cl_42_–V–Cl_43_ angles, the initial decrease with respect to the starting geometries is smaller. However, both angles continue to decrease slightly at later simulation times and reach values of 168 and 171° after 10 ps, *i.e.*, also smaller than their reference values at the ground-state minimum-energy geometry which are close to 180°.

The observations of the increase of all metal–ligand bond distances and the decrease of the trans angles can be nicely rationalized by the nature of the electronic states accessed during the dynamics. Compared to the ^3^MC_1_ ground state, that possesses the (t_2g_)^2^ configuration, the excited states ^3^MC_2_, ^3^MC_3_, and ^3^MC_4_ (which account for the majority of the population during the excited-state dynamics) are described by (t_2g_)^1^(e_g_)^1^ and (e_g_)^2^ configurations. These configurations feature electrons in the antibonding e_g_ orbitals, which are stabilized at increased metal–ligand bond distances. At the same time, the energy of these excited states decreases when the system moves further away from the ideal octahedral coordination geometry, as the reduced overlap between the metal and ligand orbitals results in a smaller ligand-field strength that decreases the energy of these excited states. Accordingly, the system moves to longer metal–ligand bond distances and smaller trans angles during the dynamics.

Fig. 5(a–i) also display the average value of the respective internal coordinates at the ISC hopping geometries as an orange line. Such geometries include both, hops from triplet to singlet states as well as hops back from singlet to triplet states. A superposition of the ISC hopping geometries is shown in Fig. 5(k). As can be seen in Fig. 5(a–c), the average V–N distances at the ISC hopping geometries are 2.34 Å for V–N_2_ and 2.28–2.29 Å for V–N_3_ and V–N_4_. These values are only reached by the ensemble of trajectories (blue curve) in the first few ps after the initial V–N bond stretching. Thus, during this time window, both ISC from the triplet to the singlet states as well as back-ISC from the singlet to the triplet states is possible. At longer simulation times, both types of ISC are quenched, which leads to the singlet population being stable after *ca.* 4 ps. The same behavior is observed for the N_3_–V–N_4_ angle in Fig. 5(g): ISC hops occur at an average angle of 157° – a value which is only realized in the ensemble of trajectories during the first few ps. At later simulation times, the N_3_–V–N_4_ angle increases, moving the system away from the ISC hopping zone and stabilizing the remaining singlet population. For the other trans angles shown in Fig. 5(h and i), the ensemble average stays close to the average of the ISC hopping geometries for longer simulation times, before finally moving away by *ca.* 4°. The V–Cl distances of the ensemble stay most of the time close to the ISC hopping averages. Only the V–Cl_41_ ensemble distance increases towards the end of the simulation time to *ca.* 2.50 Å compared to the ISC hopping average of 2.46 Å.

Finally, it is also useful to analyze the evolution of the coordinates associated with the singlet states only, green curves in Fig. 5(a–i). Note that because the majority of the population is in the triplet states at all simulation times, the averages of the exclusive triplet trajectories (not shown) closely resemble the evolution of the full ensemble. Compared to the full ensemble of trajectories (blue curve), the main difference of the V–N distances and the N_3_–V–N_4_ angle in the singlet trajectories is noticeable during the first few ps: the V–N bonds are smaller and the N_3_–V–N_4_ angle is larger than in the triplet trajectories. This is due to the fact that the majority of the singlet population is in the ^1^MC_1_ states, which are characterized by the (t_2g_)^2^ configuration with no electrons in the e_g_ orbitals. Thus, in the singlet states, the V–N distances do not tend to increase much, while keeping the N_3_–V–N_4_ angle closer to the octahedral angle of 180°. At later times, the full ensemble curve – corresponding to *ca.* 98% of triplet trajectories – resembles the average of the singlet curves, as now more and more electronic population reaches the ^3^MC_1_ triplet ground state that also possesses the (t_2g_)^2^ configuration.

## 4 Conclusion

Here we investigated the electronic excited states of the phosphorescent complex V^III^Cl_3_(ddpd) and its photodynamics using trajectory surface hopping and CASSCF potentials in 123 degrees of freedom. By analyzing the electronic wave functions and transition-density matrices, we evidence the complicated electronic structure of the MC excited states as well as the presence of excitations involving the organic ddpd ligand that requires multi-configurational methods including higher-order excitations.

Our dynamical study reveals that after excitation from the triplet ground state possessing a (t_2g_)^2^ configuration to a higher-lying MC state of (t_2g_)^1^(e_g_)^1^ character, the system evolves through different decay pathways. The main deactivation pathway involves IC to a different MC state with the (e_g_)^2^ configuration. Minor pathways include ISC to the singlet MC manifold and an additional IC pathway through triplet states involving excitations to the ddpd ligand. The time constant *τ*_ISC_ = 1.7 ± 0.3 ps obtained for the ISC in the present simulation is in good agreement with that of *τ* = 1.5 ps obtained for the rise of a long-lived signal from transient absorption experiments in the accompanying paper I.^20^ All three mechanisms end in a further triplet MC state with the (t_2g_)^1^(e_g_)^1^ configuration, which eventually relaxes back to the triplet ground state.

The dynamics is driven by elongation of all metal–ligand bond distances and a decrease of the angle between all pairs of trans-coordinated ligands further away from the octahedral 180° ideal. Both features result in a decrease of the ligand-field strength, which stabilizes the excited triplet states visited during the dynamics. The ISC leads to a temporary 10% population in the singlet states, that subsequently decreases to 1–2% at later simulation times up to 10 ps. Based on the analysis of the electronic character of all states involved in the dynamics, it is suggested that the overall population of the singlet states can be increased by increasing the ligand-field strength through suitable modification of the ligands of V^III^Cl_3_(ddpd). Such modifications are expected to result both in a more favorable population of the singlet states compared to the competing pathway to the triplet MC states, as well as a stabilization of the population in the singlet states, thus leading to larger phosphorescence yields for derivatives of V^III^Cl_3_(ddpd).

## Data availability

The data that support the findings of this study are available from the corresponding author upon reasonable request.

## Author contributions

Conceptualization, J. P. Z. and L. G.; calculations, J. P. Z. and T. K.; analysis, J. P. Z.; writing—original draft preparation, J. P. Z.; writing—review and editing, L. G.

## Conflicts of interest

There are no conflicts to declare.

## Supplementary Material

SC-012-D1SC02149D-s001

SC-012-D1SC02149D-s002

SC-012-D1SC02149D-s003
