# Supplementary material for: Ultrafast and long-time excited state kinetics of an NIR-emissive vanadium(iii) complex II. Elucidating triplet-to-singlet excited-state dynamics
Source: Chem Sci. 2021 May 26;12(32):10791–801. doi: 10.1039/d1sc02149d (PMC8372553; doi:10.1039/d1sc02149d)
Supplement: SC-012-D1SC02149D-s003 [file SC-012-D1SC02149D-s003.pdf]

Electronic Supplementary Material (ESI) for Chemical Science.
This journal is © The Royal Society of Chemistry 2021

Ultrafast and long-time excited state kinetics of an NIR-emissive vanadium(III) complex II. Elucidating Triplet-to-Singlet Excited-State Dynamics

J. Patrick Zobel,[*,†] Thomas Knoll,[†] and Leticia González[*,†,‡]

†*Institute of Theoretical Chemistry, Faculty of Chemistry, University of Vienna, Währingerstr. 19, 1090 Vienna, Austria*

‡*Vienna Research Platform on Accelerating Photoreaction Discovery, University of Vienna, Währingerstr. 19, 1090 Vienna, Austria*

E-mail: patrick.zobel@univie.ac.at; leticia.gonzalez@univie.ac.at

Supporting Information

This document contains additional data and analysis. For a structure of the document, see the following Table of Contents. Additional files containing parameters for the LVC Hamiltonian (LVC.template) and the vibrational normal modes (vcl3ddpd.molden) are provided in an additional compressed folder called lvc_data.zip.

Contents

Supporting Information **S1**

S1 Optimized Geometry **S5**

S2 CASSCF Calculations **S6**

S2.1 Active Space S6

S2.2 Number of States S9

S2.3 Characterization of the Electronic Excited States S10

S2.4 Transition Density Analysis S15

S3 Comparison to Experiment and Reference MS-CASPT2 Calculations **S17**

S4 Surface-Hopping LVC Dynamics **S22**

S4.1 Time-resolved Populations S22

S4.1.1 Spin-Adiabatic Populations S22

S4.1.2 Singlet Diabatic Populations S22

S4.2 Diabatic Character of Trajectories S24

S4.3 Kinetics S26

S4.4 Possible Influence of CASPT2 Energies on the Dynamics S27

S4.5 Triplet Density of States of Initial Conditions S28

S4.6 Normal Mode Analysis S30

S4.7 Internal Coordinate Analysis S40

S4.8 CASSCF vs. LVC Potential Energy Curves S43

References **S46**

List of Tables

S1 Ground-state geometry optimization S5

S2 Test of active spaces S8

S3 Test of number of states in the state-averaging S9

S4 Natural orbital occupation numbers S11

S5 State configurations and characterization S12

S6 Transition density matrix analysis of the T_0 . . . . S15

S7 Transition density matrix analysis of the T_1 . . . . S16

S8 Transition density matrix analysis of the T_2 . . . . S16

S9 CASSCF vs CASPT2 excitation energies in eV S19

S10 CASSCF vs CASPT2 excitation energies in cm^{-1} S20

List of Figures

S1 Active Space S7

S2 Tanabe-Sugano diagram for the d^2 Configuration S13

S3 Comparison to experiment and to CASPT2 . . . . S18

S4 Time evolution of spin-adiabatic states S22

S5 Time evolution of singlet diabatic states S23

S6 Time evolution of the diabatic electronic state character for 0-10 ps S24

S7 Time evolution of the diabatic electronic state character for 0-1 ps S25

S8 Simple kinetic model S26

S9 Triplet density of states of initial conditions S29

S10 Pyramidalization angle of the N-Me group S31

S11 Time evolution of normal mode coordinates S32

S12 Time evolution of internal coordinates S41

S13 Time evolution of internal coordinates S42

S14 CASSCF vs. LVC Potential Energy Curves S44

S1 Optimized Geometry

Table S1: Cartesian coordinates of the triplet ground-state geometry of [V(Cl_3(ddpd)] in MeCN optimized at the unrestricted B3LYP/def2-TZVP level of theory (in Å).

V	0.006707	-0.008474	0.108409	C	0.914211	4.747975	0.158015
N	0.003767	-0.003147	-2.002249	C	-0.044297	4.207536	1.014543
N	-0.158042	-2.115493	-0.103371	C	-0.383079	2.884885	0.851449
N	0.175110	2.099315	-0.091406	H	1.223431	5.779907	0.260659
C	-0.735147	-0.908846	-2.690485	H	2.152783	4.391330	-1.547013
C	-0.754985	-0.925970	-4.082013	H	-0.505111	4.793929	1.795620
C	0.006098	-0.005086	-4.777188	H	-1.096491	2.398704	1.497481
C	0.765185	0.917211	-4.081865	N	-1.508702	-1.853256	-2.010130
C	0.742987	0.902639	-2.690004	C	-2.797831	-2.218543	-2.608203
H	-1.328403	-1.675077	-4.603915	H	-2.721085	-3.042605	-3.320100
H	1.340205	1.664451	-4.604535	H	-3.478388	-2.497382	-1.807379
C	0.405431	-2.906291	0.831924	H	-3.199874	-1.345552	-3.113680
C	0.061423	-4.227582	0.994638	N	1.514346	1.848017	-2.009254
C	-0.909020	-4.760627	0.146624	C	2.798499	2.223236	-2.612194
C	-1.450704	-3.977315	-0.851266	H	2.712761	3.046744	-3.324025
C	-1.030128	-2.643845	-0.976288	H	3.479669	2.507713	-1.813944
H	-1.223089	-5.790967	0.250574	H	3.205933	1.353512	-3.119109
H	1.127635	-2.425193	1.472010	H	0.007829	-0.006316	-5.858851
H	0.527074	-4.818625	1.769333	Cl	0.004889	-0.017973	2.474462
H	-2.161849	-4.393874	-1.546122	Cl	-2.365399	0.202006	0.106489
C	1.036987	2.633800	-0.970266	Cl	2.380254	-0.213659	0.104676
C	1.450395	3.969507	-0.846619				

S2 CASSCF Calculations

S2.1 Active Space

Figure S1 shows the orbitals included in the (10,13) active space used to setup the LVC Hamiltonian. In panel (a) the orbitals are plotted with the commonly used isovalue of 0.05. As can be seen, the orbitals appear as four ligand π orbitals, 5 metal d orbitals, and 4 ligand π^* orbitals, where the π and π^* orbitals are located on the ddpd ligand. We use this orbital classification in the majority of this work. In panel (b) the same orbitals are plotted at the looser isovalue of 0.01. Interestingly, most orbitals show additional contributions not apparent before. Importantly, the five orbitals labeled as metal d orbitals show admixture of ligand orbitals, both from the π orbitals of the ddpd ligand as well as from the p orbitals of the chlorido ligands. This observation can explain the participation of the chlorido ligands in the charge flow in the excited-state dynamics discussed in the main paper. Attempts to include an extra p orbital of the chlorido ligand in the active space for the triplet states were unsuccessful, as during the optimization, the added p orbital was always rotated out of the active space to favor another ddpd π orbital. For the singlet states, the extra chlorido p orbital could be added.

The CASSCF energies for the triplet and singlet states with the different (12,14) active spaces are shown in Table S2. Adding the extra ddpd π orbital has negligible influence on the CASSCF energies of the triplet states, with maximal changes of 0.01 eV. In contrast, for the singlet states, adding the chlorido p orbital leads to lowering of all excitation energies by 0.1-0.3 eV.

(a) Orbitals in the (10,13) Active Space: Isovalue = 0.05

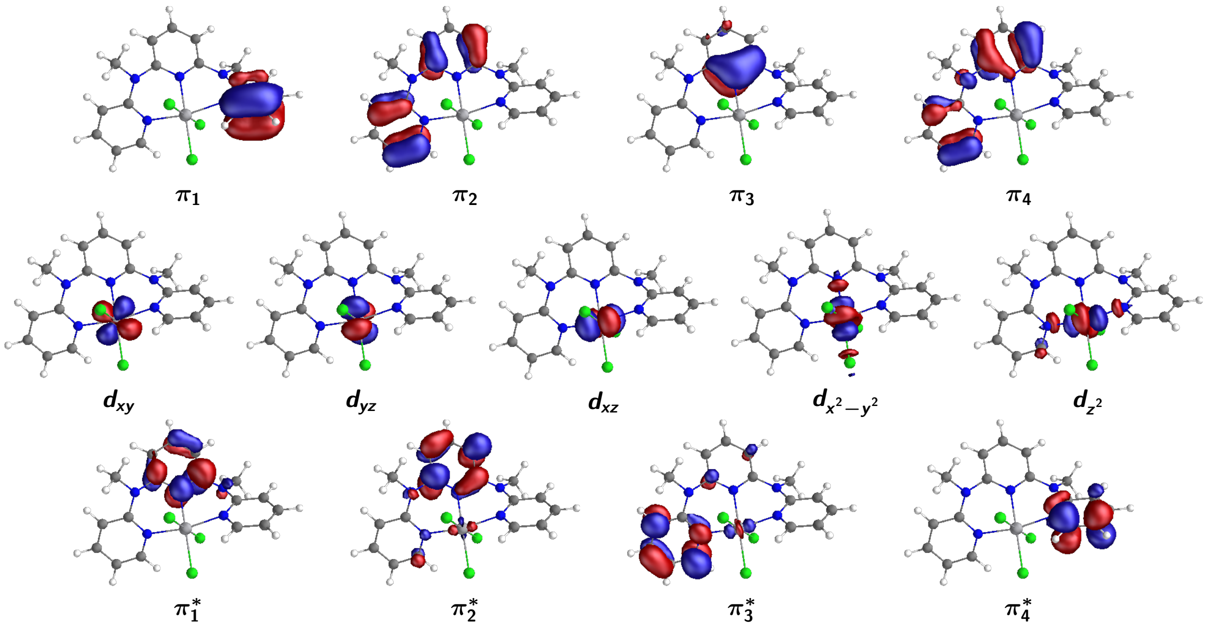

(b) Orbitals in the (10,13) Active Space: Isovalue = 0.01

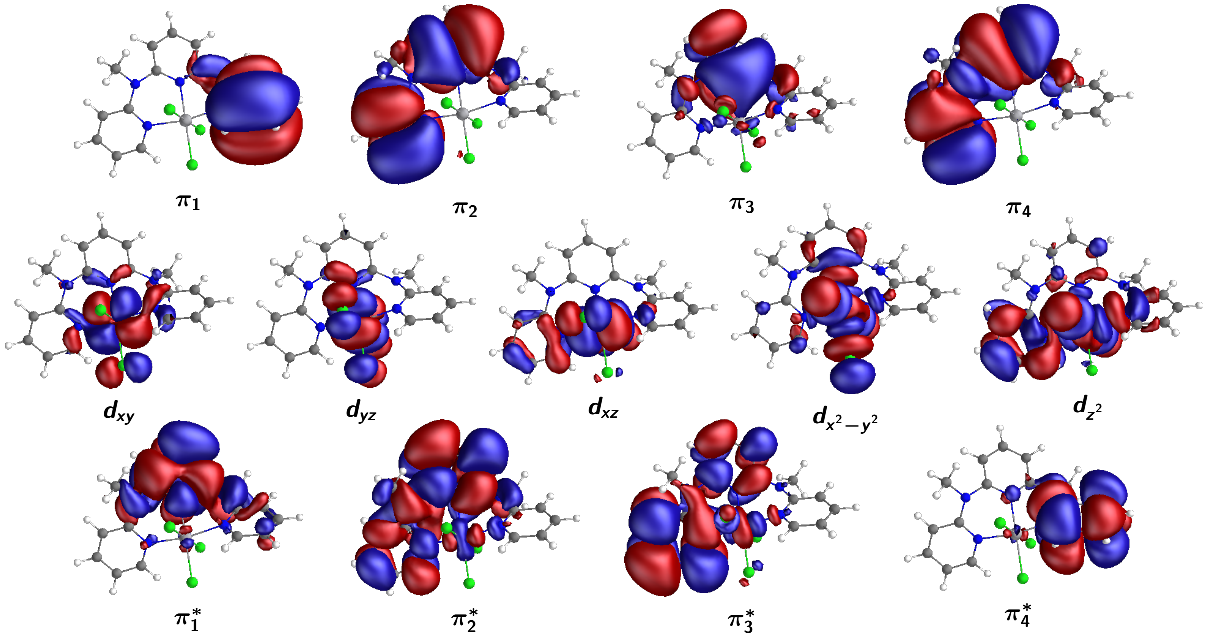

Figure S1: Orbitals included in the (10,13) active space in the CASSCF calculations. (a) Isovalue of 0.05. (b) Isovalue of 0.01

Table S2: CASSCF excitation energies in eV obtained with a (10,13) and a (12,14) active space. Orbitals of the (10,13) active space shown in Figure S1. The additional orbital in the (12,14) active space was a π orbital on the ddpd ligand for the triplet states and a p orbital on the chlorido ligand for the singlet states.

State	$E(10,13)$	$E(12,14)$	State	$E(10,13)$	$E(12,14)$
T_0 (^{3}MC$_1$)	0.00	0.00	S_1 (^{1}MC$_1$)	1.53	1.29
T_1 (^{3}MC$_1$)	0.04	0.05	S_2 (^{1}MC$_1$)	1.53	1.33
T_2 (^{3}MC$_1$)	0.10	0.10	S_3 (^{1}MC$_1$)	1.61	1.42
T_3 (^{3}MC$_2$)	1.54	1.55	S_4 (^{1}MC$_1$)	1.62	1.49
T_4 (^{3}MC$_2$)	1.70	1.70	S_5 (^{1}MC$_1$)	1.66	1.53
T_5 (^{3}MC$_2$)	1.81	1.81	S_6 (^{1}MC$_2$)	3.25	3.09
T_6 (^{3}MC$_3$)	3.16	3.17	S_7 (^{1}MC$_3$)	3.32	3.11
T_7 (^{3}MC$_3$)	3.25	3.24	S_8 (^{1}MC$_2$)	3.38	3.23
T_8 (^{3}MC$_3$)	3.36	3.36	S_9 (^{1}MC$_2$)	3.49	3.30
T_9 (^{3}MC$_4$)	3.53	3.54	S_{10} (^{1}MC$_4$)	3.71	3.54
T_{10} (^{3}DL$_1$)	4.38	4.36	S_{11} (^{1}MC$_4$)	3.81	3.71
T_{11} (^{3}DL$_1$)	4.40	4.38	S_{12} (^{1}MC$_4$)	3.91	3.86
T_{12} (^{3}DL$_1$)	4.47	4.45	S_{13} (^{1}DL$_1$)	4.39	4.19
T_{13} (^{3}DL$_2$)	4.53	4.53	S_{14} (^{1}DL$_1$)	4.43	4.28
T_{14} (^{3}DL$_2$)	4.57	4.57	S_{15} (^{1}DL$_1$)	4.48	4.33
T_{15} (^{3}DL$_2$)	4.62	4.63			

S2.2 Number of States

Using the (10,13) active space, we have performed CASSCF calculations varying the number of states included in the state averaging. The resulting energies for 12-17 triplet states and 11-16 singlet states are compiled in Table S3. As can be seen, the number of states in these ranges has only little effect on the excitation energies of all states. Furthermore, there is a pronounced energy gap between states T_{15} and T_{16} as well as between states S_{15} and S_{16} of 0.6-0.7 eV. Thus, we decided to use 16 triplet and 15 singlet states in the dynamics simulations, as these states, both, cover a sufficient energy range and are well-separated from further higher-lying excited states, that might become intruder states.

Table S3: CASSCF excitation energies in eV using a (10,13) active space and different numbers of triplet states N_T and different numbers of singlet states N_S in the state-averaging.

Triplet States							Singlet States						
N_T	12	13	14	15	16	17	N_S	11	12	13	14	15	16
T_0 (^{3}MC$_1$)	0.00	0.00	0.00	0.00	0.00	0.00	S_1 (^{1}MC$_1$)	1.56	1.50	1.51	1.51	1.53	1.54
T_1 (^{3}MC$_1$)	0.04	0.04	0.05	0.04	0.04	0.05	S_2 (^{1}MC$_1$)	1.57	1.50	1.51	1.52	1.53	1.54
T_2 (^{3}MC$_1$)	0.11	0.10	0.11	0.11	0.10	0.11	S_3 (^{1}MC$_1$)	1.65	1.58	1.59	1.59	1.61	1.61
T_3 (^{3}MC$_2$)	1.53	1.53	1.54	1.54	1.54	1.55	S_4 (^{1}MC$_1$)	1.66	1.58	1.60	1.61	1.62	1.63
T_4 (^{3}MC$_2$)	1.69	1.69	1.70	1.70	1.70	1.71	S_5 (^{1}MC$_1$)	1.69	1.62	1.65	1.66	1.66	1.67
T_5 (^{3}MC$_2$)	1.79	1.80	1.81	1.81	1.81	1.82	S_6 (^{1}MC$_2$)	3.27	3.20	3.22	3.23	3.25	3.25
T_6 (^{3}MC$_3$)	3.14	3.15	3.17	3.16	3.16	3.17	S_7 (^{1}MC$_3$)	3.34	3.27	3.29	3.31	3.32	3.33
T_7 (^{3}MC$_3$)	3.23	3.23	3.24	3.25	3.25	3.25	S_8 (^{1}MC$_2$)	3.40	3.33	3.35	3.37	3.38	3.38
T_8 (^{3}MC$_3$)	3.34	3.35	3.36	3.35	3.36	3.37	S_9 (^{1}MC$_2$)	3.51	3.44	3.47	3.48	3.49	3.49
T_9 (^{3}MC$_4$)	3.50	3.51	3.53	3.53	3.53	3.55	S_{10} (^{1}MC$_4$)	3.73	3.66	3.69	3.69	3.71	3.71
T_{10} (^{3}DL$_1$)	4.64	4.25	4.28	4.32	4.38	4.50	S_{11} (^{1}MC$_4$)	3.84	3.76	3.78	3.80	3.81	3.82
T_{11} (^{3}DL$_1$)	4.66	4.27	4.31	4.34	4.40	4.52	S_{12} (^{1}MC$_4$)		3.86	3.88	3.89	3.91	3.91
T_{12} (^{3}DL$_1$)		4.34	4.39	4.43	4.47	4.55	S_{13} (^{1}DL$_1$)			4.68	4.50	4.39	4.43
T_{13} (^{3}DL$_2$)			4.82	4.64	4.53	4.60	S_{14} (^{1}DL$_1$)				4.53	4.43	4.46
T_{14} (^{3}DL$_2$)				4.68	4.57	4.60	S_{15} (^{1}DL$_1$)					4.48	4.52
T_{15} (^{3}DL$_2$)					4.62	4.65	S_{16} (^{1}DL$_2$)						5.17
T_{16} (^{3}DL$_3$)						5.32							

S2.3 Characterization of the Electronic Excited States

The natural occupation numbers of the 16 triplet and 15 singlet states calculated with CASSCF(10,13) are collected in Table S4. These numbers allow us to assign the principal electron configuration for each state, collected in Table S5.

From Table S4 we see that, for all the triplet states, all natural orbitals posses occupation numbers close to either 0, 1, or 2, making it easy to derive a the electron configuration. In contrast, for some singlet states, some d orbitals have occupation numbers much different from 0/1/2, e.g., the S_1 state with occupation numbers of 0.69, 1.19, and 0.10 for the d_{xy}, d_{yz}, and d_{xz}. Still, as these numbers sum up close to 2, we denote the configuration as $(d_{xy,yz,xz})^2$.

As summarized in Table S5, the triplet states T_0-T_9 correspond to metal-centered states with a d_2 configuration. The triplet states T_{10}-T_{15} are characterized by a hole in one of the ligand π orbitals and an excited electron in a ligand π^* orbital, i.e., they resemble ligand-centered excited states. In the singlet manifold, states S_1-S_{12} correspond to metal-centered d^2 states, whereas S_{13}-S_{15} appear as ligand-centered excitations. We note that the states T_{10}-T_{15} and S_{13}-S_{15}, however, cannot be described simply by a $\pi \rightarrow \pi^*$ excitation as will be discussed in more detail below. The orbitals denoted in Table S5 are classified according to Figure S1(a), i.e., following only their main contribution. This neglects, e.g., contribution of the chlorido p orbitals to some of the metal d orbitals. Thus, this is only a qualitative characterization of the CASSCF results. A more quantitative description of the state characters can be obtained when analyzing the transition densities between the electronic states, as discussed in Section S2.4.

The metal-centered states can now qualitatively be classified in terms of octahedral ligand-field term symbols. For this, we show in Figure S2 the Tanabe-Sugano diagram of a d^2 configuration. We can now compare the expected order of ligand-field terms with the energies of the triplet and singlet states obtained in the CASSCF calculations (Table S2). As can be seen, the first three triplet states (T_0-T_2) are nearly degenerate with anr energetic

Table S4: Natural orbital occupation numbers of the (10,13) active space orbitals for 16 triplet and 15 singlet states.

State	π_1	π_2	π_3	π_4	d_{xy}	d_{yz}	d_{xz}	$d_{x^2-y^2}$	d_{z^2}	π_1^*	π_2^*	π_3^*	π_4^*
T_0 (^{3}MC$_1$)	1.952	1.951	1.944	1.932	0.978	0.022	0.978	0.022	0.000	0.058	0.066	0.049	0.048
T_1 (^{3}MC$_1$)	1.952	1.951	1.943	1.932	0.949	0.948	0.054	0.000	0.051	0.058	0.067	0.046	0.048
T_2 (^{3}MC$_1$)	1.952	1.951	1.944	1.932	0.022	0.978	0.979	0.000	0.022	0.058	0.066	0.049	0.048
T_3 (^{3}MC$_2$)	1.952	1.951	1.944	1.932	0.994	0.006	0.000	0.994	0.006	0.058	0.066	0.049	0.048
T_4 (^{3}MC$_2$)	1.952	1.951	1.943	1.932	0.001	0.001	0.999	0.999	0.000	0.058	0.066	0.049	0.048
T_5 (^{3}MC$_2$)	1.952	1.951	1.944	1.932	0.011	0.989	0.000	0.011	0.989	0.058	0.066	0.049	0.048
T_6 (^{3}MC$_3$)	1.952	1.950	1.943	1.932	0.074	0.926	0.000	0.926	0.075	0.058	0.067	0.049	0.048
T_7 (^{3}MC$_3$)	1.952	1.952	1.945	1.932	0.052	0.050	0.950	0.000	0.949	0.058	0.065	0.048	0.047
T_8 (^{3}MC$_3$)	1.952	1.951	1.943	1.932	0.940	0.061	0.062	0.940	0.000	0.057	0.067	0.048	0.047
T_9 (^{3}MC$_4$)	1.952	1.951	1.944	1.932	0.000	0.000	0.000	1.000	1.000	0.058	0.066	0.049	0.048
T_{10} (^{3}DL$_1$)	1.952	1.005	1.943	1.928	0.989	0.011	1.073	0.000	0.010	0.059	0.070	0.913	0.048
T_{11} (^{3}DL$_1$)	1.952	1.005	1.942	1.928	1.037	0.958	0.041	0.000	0.059	0.071	0.041	0.917	0.049
T_{12} (^{3}DL$_1$)	1.952	1.006	1.942	1.927	0.015	0.984	1.092	0.000	0.015	0.059	0.071	0.889	0.048
T_{13} (^{3}DL$_2$)	1.952	1.946	1.887	1.046	0.977	0.023	0.984	0.023	0.000	0.113	0.947	0.054	0.048
T_{14} (^{3}DL$_2$)	1.952	1.946	1.883	1.049	0.948	0.978	0.057	0.000	0.051	0.118	0.921	0.050	0.046
T_{15} (^{3}DL$_2$)	1.952	1.946	1.884	1.054	0.022	1.001	0.969	0.000	0.022	0.116	0.930	0.054	0.048
S_1 (^{1}MC$_1$)	1.952	1.952	1.932	1.944	0.694	1.196	0.105	0.005	0.001	0.057	0.066	0.048	0.048
S_2 (^{1}MC$_1$)	1.952	1.952	1.932	1.944	1.335	0.439	0.218	0.007	0.002	0.057	0.067	0.048	0.048
S_3 (^{1}MC$_1$)	1.952	1.952	1.931	1.944	1.206	0.073	0.702	0.011	0.009	0.058	0.067	0.048	0.048
S_4 (^{1}MC$_1$)	1.952	1.952	1.931	1.944	0.040	0.961	0.951	0.000	0.046	0.058	0.067	0.048	0.049
S_5 (^{1}MC$_1$)	1.952	1.952	1.931	1.944	0.009	0.952	1.016	0.008	0.014	0.058	0.067	0.048	0.048
S_6 (^{1}MC$_2$)	1.952	1.952	1.931	1.944	0.943	0.056	0.062	0.000	0.938	0.059	0.067	0.048	0.047
S_7 (^{1}MC$_3$)	1.952	1.952	1.931	1.944	0.496	0.583	0.575	0.124	0.223	0.058	0.067	0.048	0.048
S_8 (^{1}MC$_2$)	1.952	1.952	1.931	1.944	0.008	0.981	0.114	0.004	0.893	0.058	0.067	0.048	0.048
S_9 (^{1}MC$_2$)	1.952	1.952	1.931	1.944	0.061	0.062	0.939	0.940	0.000	0.057	0.067	0.048	0.047
S_{10} (^{1}MC$_4$)	1.952	1.951	1.931	1.943	0.040	0.000	0.960	0.039	0.960	0.058	0.068	0.049	0.050
S_{11} (^{1}MC$_4$)	1.953	1.952	1.932	1.945	0.004	0.996	0.004	0.996	0.000	0.057	0.066	0.047	0.047
S_{12} (^{1}MC$_4$)	1.952	1.952	1.931	1.943	0.996	0.000	0.005	0.995	0.004	0.058	0.068	0.047	0.048
S_{13} (^{1}DL$_1$)	1.952	1.952	1.045	1.889	0.992	0.978	0.022	0.000	0.021	0.111	0.941	0.048	0.048
S_{14} (^{1}DL$_1$)	1.952	1.952	1.054	1.884	0.949	0.053	0.902	0.000	0.050	0.117	0.995	0.048	0.045
S_{15} (^{1}DL$_1$)	1.952	1.952	1.062	1.886	0.021	0.961	1.022	0.021	0.000	0.115	0.912	0.048	0.048

Table S5: Configuration of the triplet and singlet states determined from natural orbital occupation numbers, characterization of d^2 states in terms of octahedral term symbols. MC denotes metal-centered states while $^{1,3}DL$ denotes d-assisted ligand-centered excitations.

	Natural Orbital Configuration			State Character	
State	π	d	π^*	Excitation	Label
T_0	$(\pi)^8$	$(d_{xy})^1(d_{xz})^1$		$(t_{2g})^2$	$^3\mathrm{MC}_1(^3T_1)$
T_1	$(\pi)^8$	$(d_{xy})^1(d_{yz})^1$		$(t_{2g})^2$	$^3\mathrm{MC}_1(^3T_1)$
T_2	$(\pi)^8$	$(d_{yz})^1(d_{xz})^1$		$(t_{2g})^2$	$^3\mathrm{MC}_1(^3T_1)$
T_3	$(\pi)^8$	$(d_{xy})^1(d_{x^2-y^2})^1$		$(t_{2g})^1(e_g)^1$	$^3\mathrm{MC}_2(^3T_2)$
T_4	$(\pi)^8$	$(d_{xz})^1(d_{x^2-y^2})^1$		$(t_{2g})^1(e_g)^1$	$^3\mathrm{MC}_2(^3T_2)$
T_5	$(\pi)^8$	$(d_{yz})^1(d_{z^2})^1$		$(t_{2g})^1(e_g)^1$	$^3\mathrm{MC}_2(^3T_2)$
T_6	$(\pi)^8$	$(d_{yz})^1(d_{x^2-y^2})^1$		$(t_{2g})^1(e_g)^1$	$^3\mathrm{MC}_3(^3T_1')$
T_7	$(\pi)^8$	$(d_{xz})^1(d_{z^2})^1$		$(t_{2g})^1(e_g)^1$	$^3\mathrm{MC}_3(^3T_1')$
T_8	$(\pi)^8$	$(d_{xy})^1(d_{x^2-y^2})^1$		$(t_{2g})^1(e_g)^1$	$^3\mathrm{MC}_3(^3T_1')$
T_9	$(\pi)^8$	$(d_{x^2-y^2})^1(d_{z^2})^1$		$(e_g)^2$	$^3\mathrm{MC}_4(^3A_2)$
T_{10}	$(\pi)^7$	$(d_{xy})^1(d_{xz})^1$	$(\pi^*)^1$	$(\pi, d \to \pi^*, d)$	$^3DL_1$
T_{11}	$(\pi)^7$	$(d_{xy})^1(d_{yz})^1$	$(\pi^*)^1$	$(\pi, d \to \pi^*, d)$	$^3DL_1$
T_{12}	$(\pi)^7$	$(d_{yz})^1(d_{xz})^1$	$(\pi^*)^1$	$(\pi, d \to \pi^*, d)$	$^3DL_1$
T_{13}	$(\pi)^7$	$(d_{xy})^1(d_{xz})^1$	$(\pi^*)^1$	$(\pi, d \to \pi^*, d)$	$^3DL_1$
T_{14}	$(\pi)^7$	$(d_{xy})^1(d_{yz})^1$	$(\pi^*)^1$	$(\pi, d \to \pi^*, d)$	$^3DL_1$
T_{15}	$(\pi)^7$	$(d_{yz})^1(d_{xz})^1$	$(\pi^*)^1$	$(\pi, d \to \pi^*, d)$	$^3DL_1$
S_1	$(\pi)^8$	$(d_{xy,yz,xz})^2$		$(t_{2g})^2$	$^1\mathrm{MC}_1(^1T_1, ^1E)$
S_2	$(\pi)^8$	$(d_{xy,yz,xz})^2$		$(t_{2g})^2$	$^1\mathrm{MC}_1(^1T_1, ^1E)$
S_3	$(\pi)^8$	$(d_{xy,xz,yz})^2$		$(t_{2g})^2$	$^1\mathrm{MC}_1(^1T_1, ^1E)$
S_4	$(\pi)^8$	$(d_{yz})^1(d_{xz})^1$		$(t_{2g})^2$	$^1\mathrm{MC}_1(^1T_1, ^1E)$
S_5	$(\pi)^8$	$(d_{xz})^1(d_{xz})^1$		$(t_{2g})^2$	$^1\mathrm{MC}_1(^1T_1, ^1E)$
S_6	$(\pi)^8$	$(d_{xz})^1(d_{z^2})^1$		$(t_{2g})^1(e_g)^1$	$^1\mathrm{MC}_2(^1T_2)$
S_7	$(\pi)^8$	$(d_{xy,yz,xz})^{1.65}(d_{x^2-y^2,z^2})^{0.35}$		$(t_{2g})^{1.65}(e_g)^{0.35}$	$^1\mathrm{MC}_3(^1A_1)$
S_8	$(\pi)^8$	$(d_{yz})^1(d_{z^2})^1$		$(t_{2g})^1(e_g)^1$	$^1\mathrm{MC}_2(^1T_2)$
S_9	$(\pi)^8$	$(d_{xz})^1(d_{x^2-y^2})^1$		$(t_{2g})^1(e_g)^1$	$^1\mathrm{MC}_2(^1T_2)$
S_{10}	$(\pi)^8$	$(d_{xz})^1(d_{z^2})^1$		$(t_{2g})^1(e_g)^1$	$^1\mathrm{MC}_4(^1T_1)$
S_{11}	$(\pi)^8$	$(d_{yz})^1(d_{x^2-y^2})^1$		$(t_{2g})^1(e_g)^1$	$^1\mathrm{MC}_4(^1T_1)$
S_{12}	$(\pi)^8$	$(d_{xy})^1(d_{x^2-y^2})^1$		$(t_{2g})^1(e_g)^1$	$^1\mathrm{MC}_4(^1T_1)$
S_{13}	$(\pi)^7$	$(d_{xy})^1(d_{xz})^1$	$(\pi^*)^1$	$(\pi, d \to \pi^*, d)$	$^1DL_1$
S_{14}	$(\pi)^7$	$(d_{xy})^1(d_{xz})^1$	$(\pi^*)^1$	$(\pi, d \to \pi^*, d)$	$^1DL_1$
S_{15}	$(\pi)^7$	$(d_{yz})^1(d_{yz})^1$	$(\pi^*)^1$	$(\pi, d \to \pi^*, d)$	$^1DL_1$

Tanabe-Sugano Diagram for the d^2 Configuration

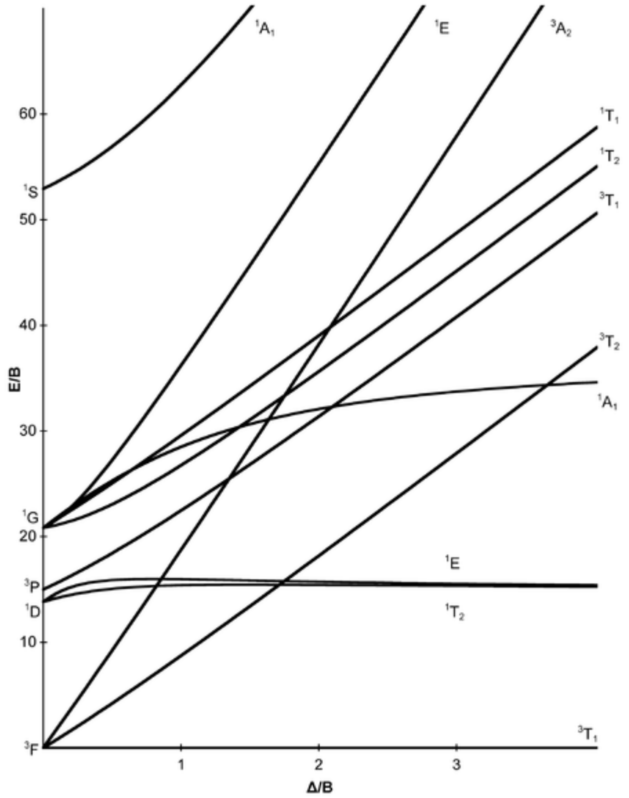

Figure S2: Tanabe-Sugano diagram for the d^2 Configuration

splitting of 0.11 eV. These states correspond to the $^3T_1$ ground-state term. At 1.54-1.81 eV, another grouping of three triplet states appears. Based on the Tanabe-Sugano diagram, these states should correspond to the $3T_2$ term (of the parent ^3F term). Next, a group of four states follows at energies 3.14-3.50 eV. Based on the Tanabe-Sugano diagram, a $3T_1$ term (of ^3P origin, threefold-degenerate) and a $^3A_1$ term (of ^3F origin, one component) is expected next. Thus, these states T_7-T_{10} likely correspond to the $^3T_1$ and $^3A_1$ terms. From Table S5, we can see that states T_7-T_9 share a $(t_{2g})^1(e_g)^1$ electron configuration, while T_{10} possesses a $(e_g)^2$ configuration. Thus, we will assign states T_7-T_9 to the $^3T1$ term whereas T_{10} corresponds to the $^3A_1$ term.

Similar to the metal-centered triplet states, we can classify the singlet states in terms of ligand-field terms. The results are shown in Table S5 alongside the results of the triplet states. Note that for the states S_1-S_5, we cannot distinguish between the components of the $^1T_1$ and ^1E terms. All calculated states share a $(t_{2g})^2$ configuration and their energetic splitting only amounts to 0.13 eV (between energies of 1.53-1.66 eV). Two of these states, the S_1 and S_2, are found at the same energy. At first, this might suggest that these two states correspond to the components of the ^1E term. However, when considering that the components all other degenerate terms are split by energies of the order of 0.1 eV, the degeneracy of the S_1 and S_2 seems coincidentally rather than as a proof of their belonging to the ^1E term.

S2.4 Transition Density Analysis

To further investigate the character of the electronic states, we have analyzed the transition densities γ_{IJ} of all states using the TheoDORE routine[1] integrated in the WFA module in OpenMolcas.[2] We thereby divided the complex $[V^{III}(Cl)_3(ddpd)]$ into three fragments: the metal atom M, the organic ddpd ligand (L1), and the three chlorido ligands (L2) [see Figure 3 in the main paper]. Here, we show the results for the transition densities of three lowest-energy triplet states T_0-T_2 with all other triplet states, i.e., γ_{IJ} with $I = 0, 1, 2$, in Tables S6-S8. Additionally, we report the weight of the sum of all single excitations $\sum\Omega$ to the CASSCF wave function in Tables S6-S8.

Table S6: Transition density matrix analysis of the transition from the T_0 to all other triplet states. $\sum\Omega$ denotes the sum of all single excitations.

γ_{IJ}		MC	ML1CT	ML2CT	L1MCT	L1C	L1L2CT	L2MCT	L2L1CT	L2C	$\sum\Omega$
T_0	T_1	0.9044	0.0186	0.0275	0.0196	0.0004	0.0005	0.0248	0.0006	0.0007	0.9972
T_0	T_2	0.8094	0.0161	0.0230	0.0201	0.0004	0.0004	0.0181	0.0006	0.0005	0.8884
T_0	T_3	0.6612	0.0247	0.0800	0.0138	0.0005	0.0006	0.0164	0.0017	0.0020	0.8008
T_0	T_4	0.8186	0.0577	0.0694	0.0196	0.0014	0.0012	0.0169	0.0017	0.0014	0.9878
T_0	T_5	0.2249	0.0269	0.0075	0.0051	0.0006	0.0010	0.0082	0.0002	0.0003	0.2745
T_0	T_6	0.3831	0.0187	0.0316	0.0083	0.0004	0.0006	0.0120	0.0007	0.0011	0.4564
T_0	T_7	0.7857	0.0632	0.0561	0.0190	0.0015	0.0013	0.0162	0.0014	0.0011	0.9455
T_0	T_8	0.5547	0.0600	0.0175	0.0119	0.0012	0.0015	0.0135	0.0004	0.0004	0.6610
T_0	T_9	0.0555	0.0053	0.0035	0.0009	0.0000	0.0002	0.0015	0.0001	0.0001	0.0671
T_0	T_{10}	0.0000	0.0003	0.0000	0.0096	0.4653	0.0016	0.0000	0.0022	0.0000	0.4791
T_0	T_{11}	0.0000	0.0001	0.0000	0.0057	0.0154	0.0001	0.0000	0.0002	0.0000	0.0215
T_0	T_{12}	0.0000	0.0001	0.0000	0.0006	0.0091	0.0000	0.0000	0.0001	0.0000	0.0098
T_0	T_{13}	0.0000	0.0010	0.0000	0.0012	0.4853	0.0005	0.0000	0.0004	0.0000	0.4884
T_0	T_{14}	0.0000	0.0014	0.0000	0.0002	0.0020	0.0000	0.0000	0.0000	0.0000	0.0037
T_0	T_{15}	0.0000	0.0013	0.0000	0.0000	0.0001	0.0000	0.0000	0.0000	0.0000	0.0015

Table S7: Transition density matrix analysis of the transition from the T_1 to all other triplet states. $\sum\Omega$ denotes the sum of all single excitations.

γ_{IJ}		MC	ML1CT	ML2CT	L1MCT	L1C	L1L2CT	L2MCT	L2L1CT	L2C	$\sum\Omega$
T_1	T_0	0.9044	0.0196	0.0248	0.0186	0.0004	0.0006	0.0275	0.0005	0.0007	0.9972
T_1	T_2	0.9056	0.0211	0.0238	0.0236	0.0004	0.0005	0.0214	0.0006	0.0006	0.9976
T_1	T_3	0.8181	0.0294	0.0994	0.0095	0.0003	0.0009	0.0266	0.0011	0.0033	0.9887
T_1	T_4	0.0414	0.0029	0.0034	0.0028	0.0002	0.0002	0.0035	0.0002	0.0003	0.0549
T_1	T_5	0.8171	0.0903	0.0344	0.0251	0.0028	0.0014	0.0128	0.0010	0.0006	0.9854
T_1	T_6	0.7971	0.0335	0.0891	0.0242	0.0010	0.0006	0.0134	0.0028	0.0014	0.9631
T_1	T_7	0.1673	0.0058	0.0059	0.0053	0.0003	0.0003	0.0064	0.0002	0.0003	0.1920
T_1	T_8	0.8001	0.0900	0.0296	0.0101	0.0010	0.0029	0.0254	0.0004	0.0009	0.9604
T_1	T_9	0.0428	0.0037	0.0031	0.0009	0.0001	0.0001	0.0010	0.0001	0.0001	0.0518
T_1	T_{10}	0.0000	0.0001	0.0000	0.0013	0.0140	0.0000	0.0000	0.0001	0.0000	0.0155
T_1	T_{11}	0.0000	0.0004	0.0000	0.0070	0.4729	0.0017	0.0000	0.0022	0.0000	0.4842
T_1	T_{12}	0.0000	0.0001	0.0000	0.0005	0.0016	0.0000	0.0000	0.0000	0.0000	0.0023
T_1	T_{13}	0.0000	0.0002	0.0000	0.0010	0.0020	0.0000	0.0000	0.0000	0.0000	0.0033
T_1	T_{14}	0.0000	0.0016	0.0000	0.0015	0.4860	0.0004	0.0000	0.0004	0.0000	0.4900
T_1	T_{15}	0.0000	0.0004	0.0000	0.0000	0.0000	0.0000	0.0000	0.0000	0.0000	0.0004

Table S8: Transition density matrix analysis of the transition from the T_2 to all other triplet states. $\sum\Omega$ denotes the sum of all single excitations.

γ_{IJ}		MC	ML1CT	ML2CT	L1MCT	L1C	L1L2CT	L2MCT	L2L1CT	L2C	$\sum\Omega$
T_2	T_0	0.8094	0.0201	0.0181	0.0161	0.0004	0.0006	0.0230	0.0004	0.0005	0.8884
T_2	T_1	0.9056	0.0236	0.0214	0.0211	0.0004	0.0006	0.0238	0.0005	0.0006	0.9976
T_2	T_3	0.1941	0.0061	0.0240	0.0065	0.0002	0.0002	0.0051	0.0008	0.0006	0.2377
T_2	T_4	0.8150	0.0574	0.0689	0.0149	0.0010	0.0016	0.0226	0.0012	0.0019	0.9846
T_2	T_5	0.6331	0.0685	0.0286	0.0131	0.0013	0.0018	0.0163	0.0006	0.0007	0.7641
T_2	T_6	0.5910	0.0198	0.0654	0.0123	0.0004	0.0005	0.0155	0.0013	0.0017	0.7080
T_2	T_7	0.8115	0.0653	0.0575	0.0153	0.0012	0.0018	0.0225	0.0011	0.0016	0.9778
T_2	T_8	0.4054	0.0364	0.0167	0.0112	0.0010	0.0009	0.0103	0.0004	0.0004	0.4829
T_2	T_9	0.0309	0.0017	0.0033	0.0009	0.0000	0.0000	0.0006	0.0001	0.0001	0.0375
T_2	T_{10}	0.0000	0.0000	0.0000	0.0017	0.0101	0.0000	0.0000	0.0001	0.0000	0.0119
T_2	T_{11}	0.0000	0.0000	0.0000	0.0055	0.0006	0.0000	0.0000	0.0001	0.0000	0.0064
T_2	T_{12}	0.0000	0.0004	0.0000	0.0102	0.4787	0.0017	0.0000	0.0023	0.0000	0.4933
T_2	T_{13}	0.0000	0.0000	0.0000	0.0009	0.0002	0.0000	0.0000	0.0000	0.0000	0.0012
T_2	T_{14}	0.0000	0.0000	0.0000	0.0003	0.0000	0.0000	0.0000	0.0000	0.0000	0.0003
T_2	T_{15}	0.0000	0.0018	0.0000	0.0017	0.4879	0.0004	0.0000	0.0004	0.0000	0.4923

S3 Comparison to Experiment and Reference MS-CASPT2 Calculations

Here we shall discuss the CASSCF excitation energies of $[V^{III}(Cl)_3(ddpd)]$ in MeCN against experiment as well as against benchmark MS-CASPT2 calculations.

Figure S3(a) shows the CASSCF excitation energies together with the experimental absorption spectrum. Experimentally (blue line), there is a small band with a maximum at 2.65 eV, followed by a larger band peaking around 3.85 eV. The vertical CASSCF excitations at the equilibrium geometry (red sticks) predict the first absorbing state at 1.70 eV, followed by a state with larger oscillator strength at 3.25 eV. The LVC spectrum (green line) –based on the convolution of LVC-CASSCF excitation energies of 20000 geometries obtained from a Wigner sampling– predicts two bands with maxima at 1.82 and 3.26 eV, i.e., very close to the CASSCF excitation energies at the FC geometry, but considerably red-shifted (0.6 and 1.0 eV) from the experiment. These results are in agreement with the common acceptance that CASSCF wave functions can provide qualitative correct descriptions of a multi-configurational system, but CASSCF energies can inherently be subject to noticeable errors.[3]

Accordingly, the validity of the CASSCF calculations is also assessed against the more accurate MS-CASPT2 procedure. One important parameter in MS-CASPT2 calculations is the so-called IPEA shift ε.[4] While in organic molecules it has been shown[5] that one should forego the use of the IPEA shift, in transition metal complexes a recommendation is missing. Therefore, we performed MS-CASPT2 calculation with the historically recommended[4] IPEA value of $\varepsilon = 0.25$ a.u. (labelled as CASPT2-IPEA) and without, $\varepsilon = 0$ (CASPT2-NOIPEA). Tables S9 and S10 list the two sets of obtained MS-CASPT2 energies of the triplet and singlet states alongside the CASSCF(10,13) reference as well as the CASSCF-CASPT2 energy differences in eV and cm^{-1}, respectively. Additionally, the results are pictorially shown in Figure S3(b).

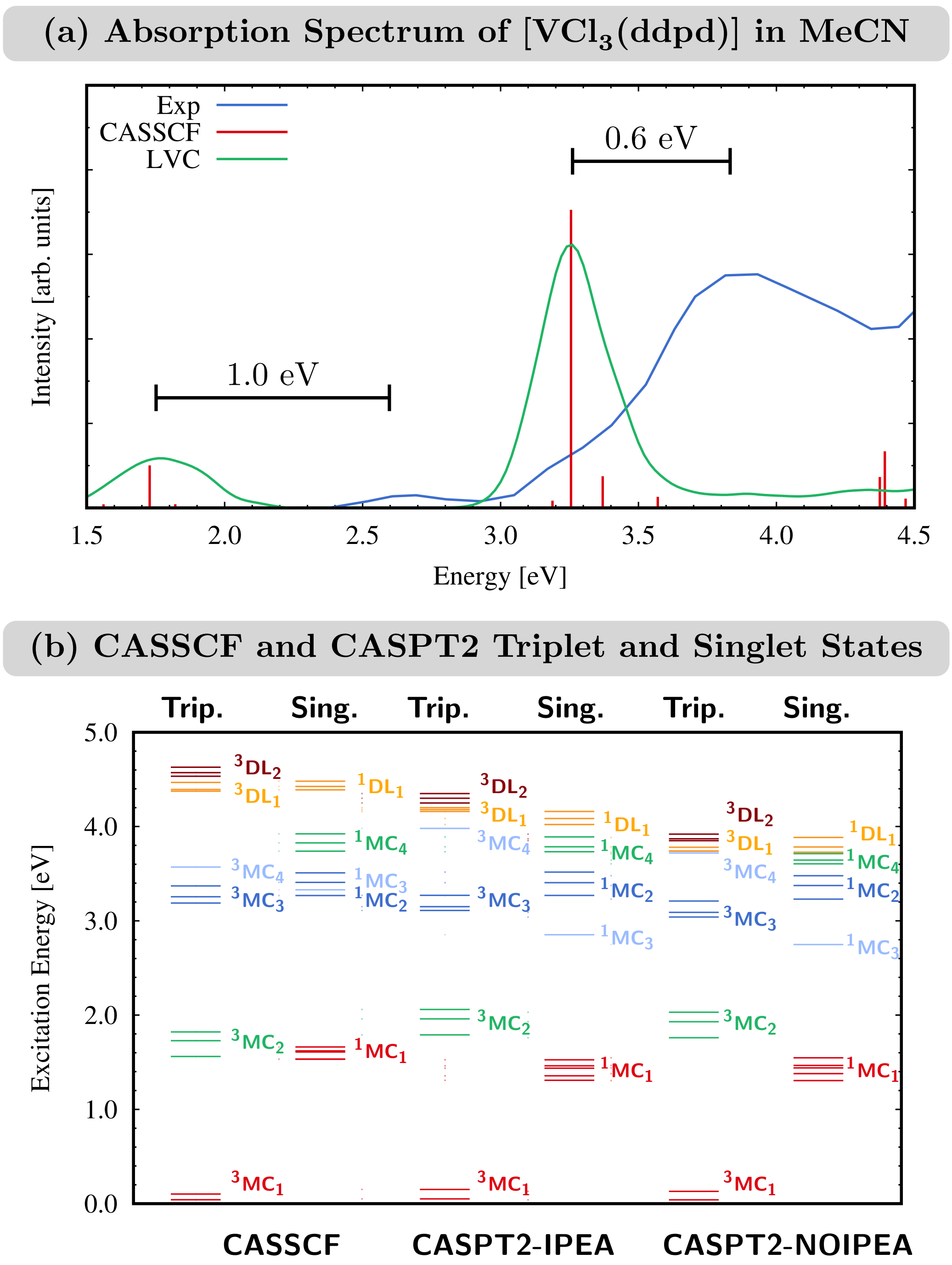

Figure S3: (a) Experimental absorption spectrum of $[V^{III}(Cl)_3(ddpd)]$ in MeCN (blue line), CASSCF excited states at the equilibrium in MeCN (red sticks), and spectrum calculated using the LVC model (green line). (b) Excitation energies (in eV) calculated with CASSCF, CASPT2-IPEA (ε = 0.25 a.u.) and CASPT2-NOIPEA (ε = 0). ε denotes the IPEA shift parameter. States are labelled and color-coded according to their state character as introduced in Figure 2 in the main paper.

Table S9: Excitation energies in eV computed at the CASSCF and MS-CASPT2 levels of theory using a (10,13) active space and CASSCF-CASPT2 energy differences. MS-CASPT2 calculations are conducted with IPEA shift values of 0 ("NOIPEA") and 0.25 a.u. ("IPEA").

State	E_{CASSCF}	E^{IPEA}_{CASPT2}	E^{NOIPEA}_{CASPT2}	ΔE^{IPEA}_{CASPT2}	$\Delta E^{NOIPEA}_{CASPT2}$
$^3MC_1$ (T_0)	0.00	0.00	0.00	0.00	0.00
$^3MC_1$ (T_1)	0.04	0.05	0.03	-0.01	0.00
$^3MC_1$ (T_2)	0.09	0.14	0.13	-0.04	-0.03
$^3MC_2$ (T_3)	1.54	1.78	1.76	-0.24	-0.21
$^3MC_2$ (T_4)	1.70	1.95	1.93	-0.25	-0.23
$^3MC_2$ (T_5)	1.80	2.06	2.02	-0.25	-0.22
$^3MC_3$ (T_6)	3.16	3.11	3.04	0.05	0.12
$^3MC_3$ (T_7)	3.24	3.14	3.09	0.10	0.15
$^3MC_3$ (T_8)	3.35	3.26	3.20	0.09	0.15
$^3MC_4$ (T_9)	3.53	3.98	3.71	-0.44	-0.18
$^3DL_1$ (T_{10})	4.37	4.16	3.73	0.21	0.63
$^3DL_1$ (T_{11})	4.39	4.18	3.74	0.21	0.65
$^3DL_1$ (T_{12})	4.46	4.20	3.78	0.26	0.68
$^3DL_2$ (T_{13})	4.53	4.25	3.84	0.27	0.68
$^3DL_2$ (T_{14})	4.56	4.29	3.86	0.27	0.69
$^3DL_2$ (T_{15})	4.62	4.34	3.92	0.27	0.69
$^1MC_1$ (S_1)	1.52	1.30	1.30	0.22	0.22
$^1MC_1$ (S_2)	1.53	1.35	1.37	0.17	0.15
$^1MC_1$ (S_3)	1.60	1.43	1.44	0.16	0.16
$^1MC_1$ (S_4)	1.61	1.46	1.46	0.15	0.15
$^1MC_1$ (S_5)	1.65	1.52	1.54	0.13	0.10
$^1MC_2$ (S_6)	3.25	3.26	3.23	-0.01	0.02
$^1MC_2$ (S_8)	3.38	3.40	3.37	-0.02	0.01
$^1MC_2$ (S_9)	3.49	3.51	3.47	-0.02	0.02
$^1MC_3$ (S_7)	3.32	2.85	2.74	0.47	0.58
$^1MC_4$ (S_{10})	3.70	3.73	3.60	-0.02	0.10
$^1MC_4$ (S_{11})	3.81	3.78	3.64	0.02	0.16
$^1MC_4$ (S_{12})	3.90	3.88	3.71	0.01	0.19
$^1DL_1$ (S_{13})	4.39	4.02	3.72	0.37	0.66
$^1DL_1$ (S_{14})	4.42	4.08	3.78	0.34	0.64
$^1DL_1$ (S_{15})	4.48	4.16	3.88	0.32	0.59

Table S10: Excitation energies in cm^{-1} computed at the CASSCF and MS-CASPT2 levels of theory using a (10,13) active space and CASSCF-CASPT2 energy differences. MS-CASPT2 calculations are conducted with IPEA shift values of 0 ("NOIPEA") and 0.25 a.u. ("IPEA").

State	E_{CASSCF}	E_{CASPT2}^{IPEA}	E_{CASPT2}^{NOIPEA}	ΔE_{CASPT2}^{IPEA}	$\Delta E_{CASPT2}^{NOIPEA}$
$^3MC_1$ (T_0)	0	0	0	0	0
$^3MC_1$ (T_1)	332	437	282	-104	50
$^3MC_1$ (T_2)	799	1171	1083	-371	-283
$^3MC_2$ (T_3)	12460	14431	14214	-1970	-1753
$^3MC_2$ (T_4)	13731	15795	15598	-2063	-1866
$^3MC_2$ (T_5)	14577	16639	16372	-2061	-1794
$^3MC_3$ (T_6)	25505	25100	24533	404	972
$^3MC_3$ (T_7)	26174	25368	24936	806	1238
$^3MC_3$ (T_8)	27088	26341	25858	746	1230
$^3MC_4$ (T_9)	28507	32110	30003	-3603	-1495
$^3DL_1$ (T_{10})	35303	33565	30152	1737	5151
$^3DL_1$ (T_{11})	35453	33746	30180	1706	5273
$^3DL_1$ (T_{12})	36046	33910	30495	2136	5551
$^3DL_2$ (T_{13})	36546	34314	31025	2232	5521
$^3DL_2$ (T_{14})	36848	34656	31209	2191	5639
$^3DL_2$ (T_{15})	37285	35066	31640	2218	5644
$^1MC_1$ (S_1)	12329	10550	10534	1779	1795
$^1MC_1$ (S_2)	12345	10935	11125	1409	1219
$^1MC_1$ (S_3)	12958	11589	11616	1368	1341
$^1MC_1$ (S_4)	13039	11793	11823	1246	1216
$^1MC_1$ (S_5)	13362	12309	12478	1053	884
$^1MC_2$ (S_6)	26221	26369	26054	-148	167
$^1MC_2$ (S_8)	27275	27457	27215	-182	60
$^1MC_2$ (S_9)	28167	28361	28053	-194	114
$^1MC_3$ (S_7)	26783	23007	22167	3776	4616
$^1MC_4$ (S_{10})	29913	30108	29074	-194	838
$^1MC_4$ (S_{11})	30759	30532	29389	227	1370
$^1MC_4$ (S_{12})	31509	31374	29947	134	1561
$^1DL_1$ (S_{13})	35428	32435	30068	2993	5359
$^1DL_1$ (S_{14})	35720	32947	30517	2772	5203
$^1DL_1$ (S_{15}v	36149	33555	31327	2594	4822

The largest difference between CASSCF and both sets of MS-CASPT2 results are found for the ^{3}MC$_4$ and ^{1}MC$_3$ states as well as for the ^{3}DL$_{1,2}$ and ^{1}DL$_1$ states. We recall that the former two are doubly excited states characterized by a $(e_g)^2$ configuration, while the latter two possess 50 % double-excitation character. Both ^{3}DL$_{1,2}$ and ^{1}DL$_1$ states are red-shifted when going from CASSCF to MS-CASPT2. Among the ^{3}MC$_4$ and ^{1}MC$_3$ states, only the ^{1}MC$_3$ states is red-shifted when going from CASSCF to MS-CASPT2, while the ^{3}MC$_4$ is found at higher energies with MS-CASPT2. Thus, apart from the magnitude of the energetic shift, there is no common trend for states with significant double-excitation character when going from CASSCF to MS-CASPT2.

A more specific difference between CASSCF and MS-CASPT2 results can be found for the ^{3}MC$_2$ and ^{1}MC$_1$ states –that take the role of lowest-excited states of each spin multiplicity when taking the ^{3}MC$_1$ states as the ground state. The ^{1}MC$_1$ states are found at lower energies while the ^{3}MC$_2$ states are found at higher energies when going from CASSCF to MS-CASPT2. In addition, we also note that the ^{1}MC$_3$ state is stabilized in energy when going from CASSCF to IPEA-CASPT2 and NOIPEA-CASPT2 by 0.47 and 0.58 eV, respectively. Interestingly, we find that IPEA-CASPT2 predicts larger excitation energies compared to NOIPEA-CASPT2, as it was found for organic molecules.[5] These differences are of the order of 0.1 eV for the MC states, while they can be 0.4-0.5 eV for the DL states.

While it is computationally unfeasible to perform the LVC parameterization using the CASPT2 level of theory, we will discuss later the possible implications on the excited-state dynamics of the here observed differences between CASSCF and CASPT2 results.

S4 Surface-Hopping LVC Dynamics

S4.1 Time-resolved Populations

S4.1.1 Spin-Adiabatic Populations

In Figure S4, we show the time evolution of the spin-adiabatic triplet and singlet states. Spin-adiabatic states are states characterized by their spin value and their energetic ordering. This representation is complementary to the diabatic representation (see Fig. 4 in the main paper), in which the states can be distinguished by their electronic character.

Time Evolution of Spin-Adiabatic Electronic State Populations

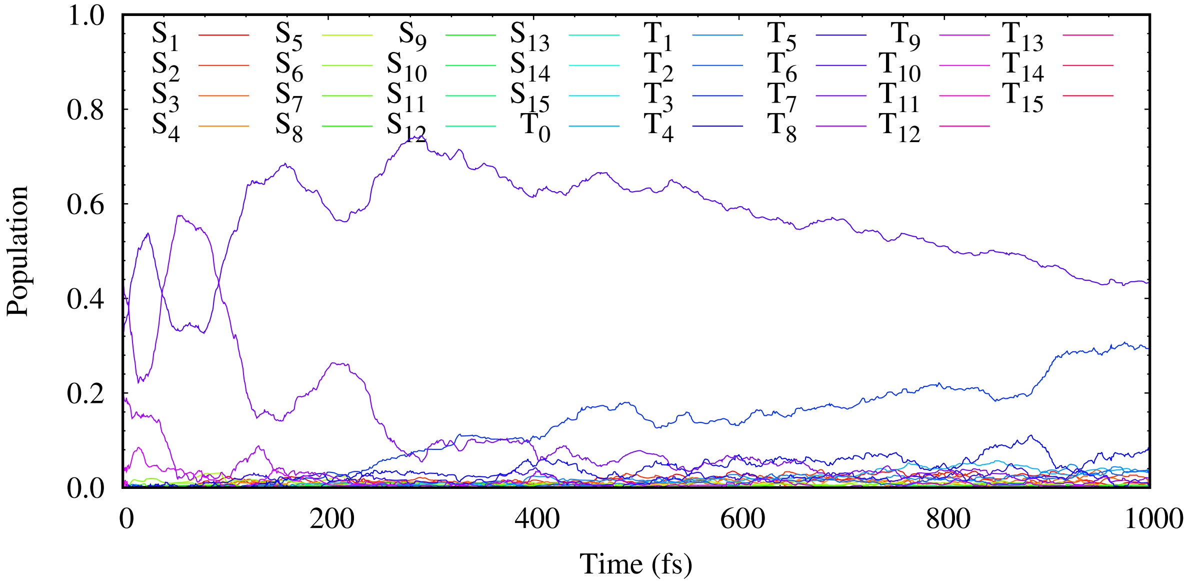

Figure S4: Time-evolution of spin-adiabatic electronic states from the LVC dynamics of $[V^{III}(Cl)_3(ddpd)]$ started at an excitation energy range between 3.0-3.5 eV.

S4.1.2 Singlet Diabatic Populations

In Figure S5, we show the time evolution of the diabatic singlet state populations, where the states are characterized according to Table S5. As can be seen, except for early simulation times, the singlet population is almost completely in the $^1MC_1$ states, which correspond to the $^1T_2$ and ^1E components of the $(t_{2g})^2$ configuration.

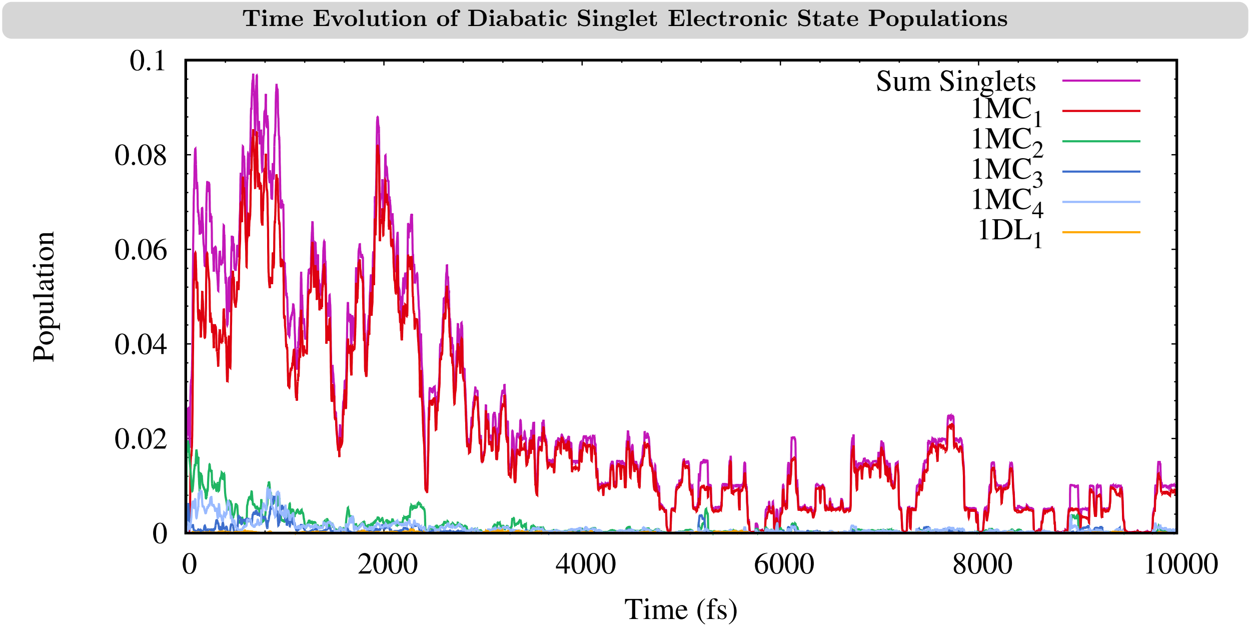

Figure S5: Time evolution of diabatic singlet electronic states from the LVC dynamics of [$V^{III}(Cl)_3$(ddpd)]started at an excitation energy range between 3.0-3.5 eV.

S4.2 Diabatic Character of Trajectories

In the main paper, we show the diabatic character of sample trajectories for simulation times up to 1 ps (Figure 4). Figure S6 displays the time evolution up to 10 ps. In both Figures,

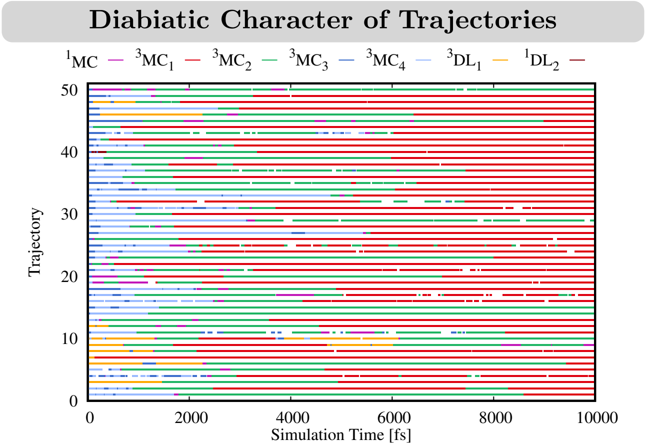

Figure S6: Time evolution of the diabatic electronic state character for sample trajectories from the LVC dynamics of [$V^{III}(Cl)_3$(ddpd)] after excitation between 3.0-3.5 eV for the time window 0-10 ps. Characterization using a threshold of $c^2 = 0.6$.

we assigned a diabatic state character to a trajectory, if its diabatic population of a specific character exceeded a threshold of $c^2 = 0.6$. The influence of this threshold is analysed in Figure S7, we we compare results for 1 ps using the stricter threshold of $c^2 = 0.7$. As can be seen, using $c^2 = 0.7$, the amount of time steps where a trajectory cannot be assigned to a specific diabatic states increases, i.e., from 5 % to 13 % of the steps. However, overall, no large differences are apparent when comparing both thresholds. Thus, we used the threshold $c^2 = 0.6$ in the main paper.

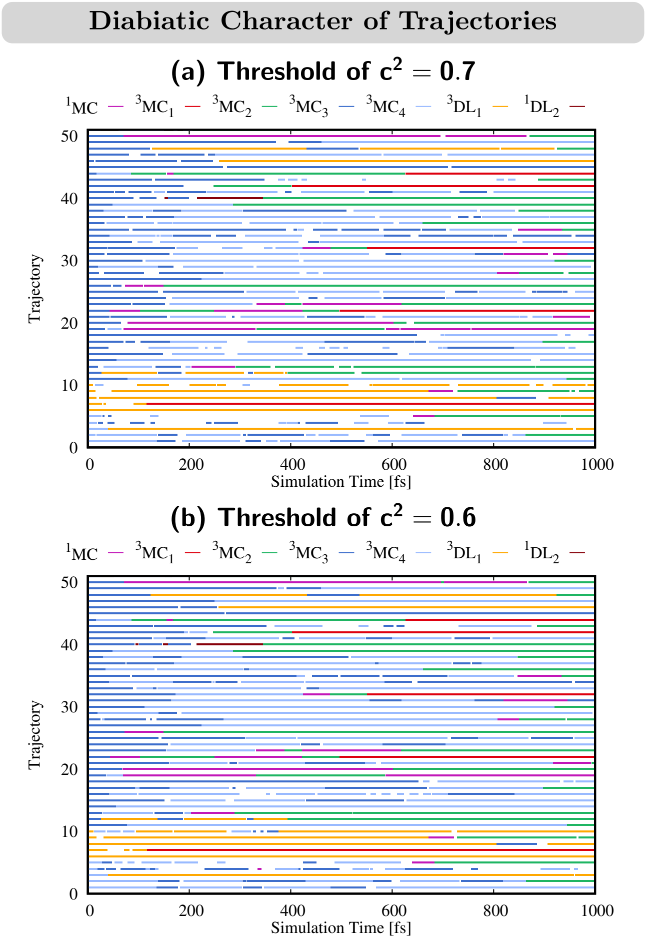

Figure S7: Time evolution of the diabatic electronic state character for sample trajectories from the LVC dynamics of $[V^{III}(Cl)_3(ddpd)]$after excitation between 3.0-3.5 eV for the time window 0-1 ps. Characterization using a threshold of $c^2 = 0.7$ for (a) and $c^2 = 0.6$ for (b), respectively.

S4.3 Kinetics

In Figure S8, we show the results of the kinetic model based on eqs. 4-6 from the main paper. For early simulation times, the fit for the $^3MC_3$ state populations decreases too rapidly compared to the actual $^3MC_3$ population. In addition, the fit of the $^3MC_2$ population decreases too rapidly at later simulation times. For these reasons, the decays of both populations were rather described by a biexponential possessing a fast and slow component, as discussed in the main paper.

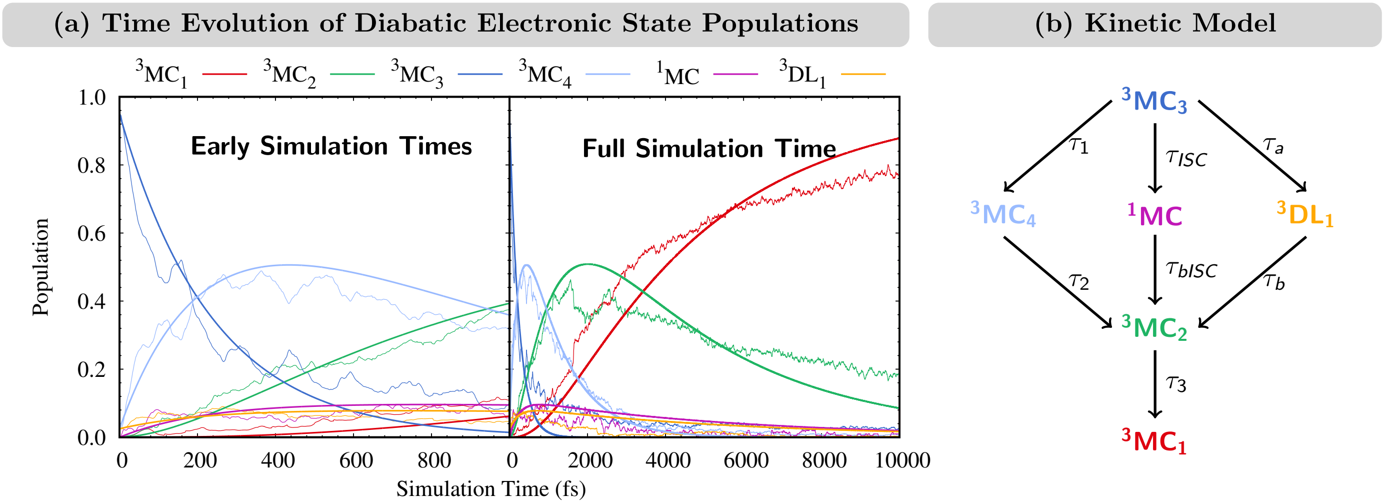

Figure S8: (a) Time evolution of the diabatic electronic state populations. Thin lines represent state populations while thick lines represent fitted curves. (b) Kinetic model used for the fitting.

S4.4 Possible Influence of CASPT2 Energies on the Dynamics

The dynamics simulations presented in he main paper are based on LVC potentials parameterized at the CASSCF level of theory. Unfortunately, it is computationally unfeasible to perform this parameterization at the MS-CASPT2 level of theory; however, here we would like to discuss how the found differences between the CASSCF and MS-CASPT2 excited state energies at the equilibrium geometry (recall Section S3) could affect the non-adiabatic dynamics.

First, we noted that the $^3MC_4$ state appears at higher energies at the CASPT2 level of theory. This would likely quench the main relaxation pathway $^3MC_3 \rightarrow ^3MC_4$ to some extent, channeling more population through the $^3MC_3 \rightarrow ^3DL_1$ and $^3MC_3 \rightarrow ^1MC$ pathways. Second, we noted that the lowest-excited singlet state, $^1MC_1$, which is also the state that accounts for the majority of the total singlet population, is lowered in energy at the MS-CASPT2 level of theory, while the lowest-excited triplet state, $^3MC_2$, is found at higher energies. Since, in our kinetic model the ^1MC states are partly depopulated to the $^3MC_2$ state via back-ISC, the energetic shift when going from CASSCF to MS-CASPT2 could quench this depopulation. Third, since the $^1MC_3$ state is stabilized by ca. 0.5 eV at the MS-CASPT2 level of theory, it may play a larger role in the dynamics leading to an overall larger singlet population. Thus, we can presume that, both, the fraction of population initially undergoing ISC from the $^3MC_4$ to the ^1MC states, as well as the fraction of population then staying in the ^1MC states are underestimated in the CASSCF-based dynamics. Accordingly, the present results serve only to establish lower bounds for these quantities, and the true yield of singlet population leading to the experimentally observed phosphorescence can be larger.

S4.5 Triplet Density of States of Initial Conditions

The majority of the electronic population in the dynamics simulation is initially excited in the $^3MC_3$ and $^3MC_4$ states (97.5 %), with the remaining population being excited in the $^3DL_1$ states (2.5 %). According to the energies of these states at the FC geometry (Figure 2 in the main paper, Table S3 in the SI), the $^3DL_1$ states lie ca. 1 eV higher in energy than the $^3MC_{3,4}$ states. Nevertheless, the dynamics simulation show population flow from the $^3MC_3$ to the $^3DL_1$ states which increases the population of these states temporarily up to ca. 10 % (Figure 4 in the main paper). This population flow is enabled due to the broad density of states of the $^3DL_1$ states. To demonstrate this, we show in Figure S9 the density of states (DOS) of the individual triplet states at the geometries considered in the initial conditions –these are shown alongside as a superposition in Figure S9. As can be seen, the density of the ^3DL states (negative axis) is considerably broader than that of the ^3MC states. For the $^3DL_1$ states, this DOS spans an interval of ca. 2 eV (from ca. 3 to 5 eV). In contrast, the spread of the DOS of the $^3MC_3$ states, for example, is much smaller, with most DOS between 3.0 and 3.5 eV. The larger spread of the DOS of the ^3DL states can be understood by considering the geometries included in the initial conditions (right-hand side of Figure S9). As can be seen, the geometries deviate from FC geometry primarily through motion in the ddpd ligand, while, e.g., the V−X distances to the ligating atoms (X = N, Cl) coordinated to the central V atom as well as their bonding angles X−V−Y remain almost fixed (X, Y, = N, Cl). As the ^3DL states are described in large parts by $\pi \rightarrow \pi^*$ excitations at the ddpd ligand, the energy of these states is varied already in the initial conditions, i.e., geometries that are sampled from a Wigner distribution around the FC geometry. In contrast, the ^3MC are not affected by the motion in the ligand. They will only be influenced by the dynamics in the excited states which will change the V−X bond distances and X−V−Y bond angles as is discussed in Section 3.2 in the main paper.

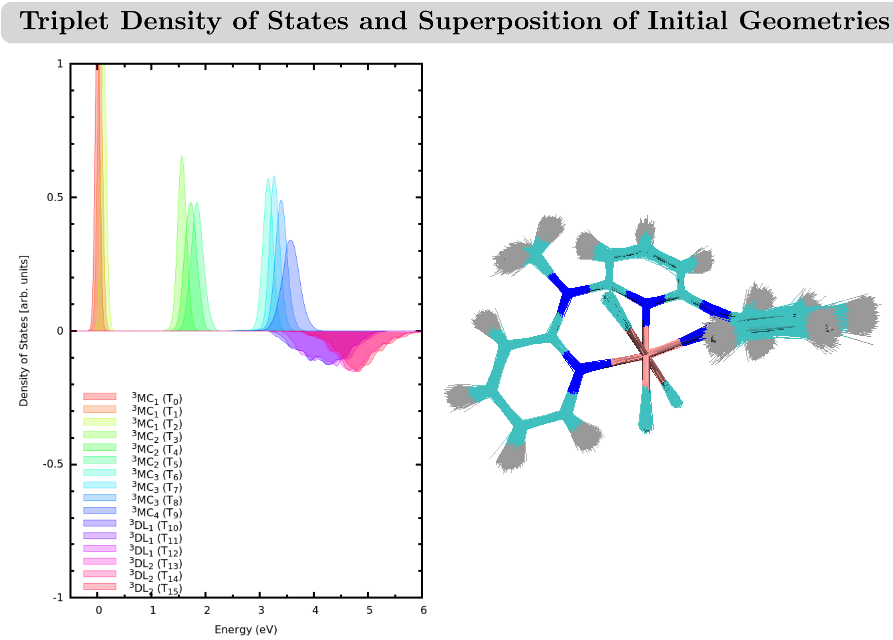

Figure S9: Left-hand side: Density of states of triplet MC states (positive axis) and triplet DL states (negative axis) of the 20000 geometries considered in the initial conditions of the dynamics. Right-hand side: superposition of the geometries of the initial conditions.

S4.6 Normal Mode Analysis

Figure S11 displays the time-evolution of all 123 normal modes of $[V^{III}(Cl)_3(ddpd)]$; thick lines represent the average of the normal mode coordinates for the ensemble of 200 trajectories and the gray shaded area is the standard deviation. Their analysis allows us identifying the most important normal modes responsible for the excited-state dynamics, as those whose average change the most over the course of the simulation time. This includes the following normal modes: 7, 9, 12, 17, 21, 27, 28, 32, 34, 36, 38, 39, 46, 48, 69, and 71. These modes are distinctly delocalized over the whole complex but feature movements of the V-Cl bonds, stretching of the V-N bonds, deformation of the X-V-Y bond angles (where X and Y are either Cl or N), and a wagging motion of the methyl groups at the N-bridges between the pyridine rings.

The time evolution of the internal coordinates V-X bond stretches and X-V-Y bond angles are discussed in the main paper. Here we comment on the wagging motion of the methyl groups, which is less important. To analyze the wagging motion of the methyl groups, we followed the time evolution of different pyramidalization angles including the methyl N atoms, the methyl C atom, and the two carbon atoms from the pyridine units that are bound to the respective nitrogen atom. For a combination of atoms A-B-C-D, the pyramidalization angle p is defined as 90° minus the angle between the bond of A-B and the normal vector of the plane B-C-D. The time evolution of the so-calculated angles p are shown in Figure S10. As can be seen, all remain close to the reference value of the ground-state geometry.

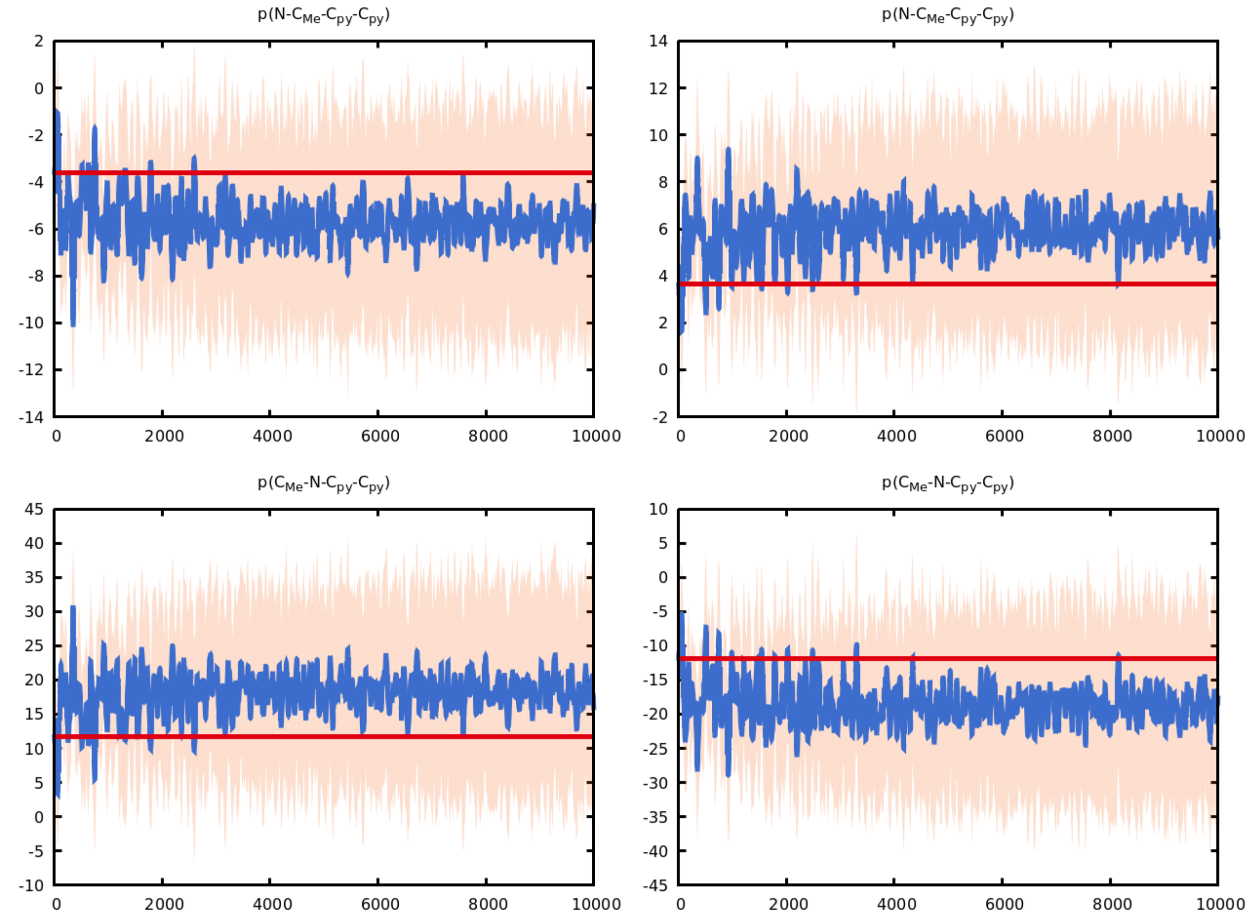

Figure S10: Time evolution of the pyramidalization angle of the N-Me group according to the different definitions described in the text part. Average (blue line) and standard deviation (orange filled area) of 200 trajectories and reference values (red line) of the FC geometry.

Figure S11: Time evolution of each normal mode coordinate as an average (plus standard deviation) over 200 trajectories.

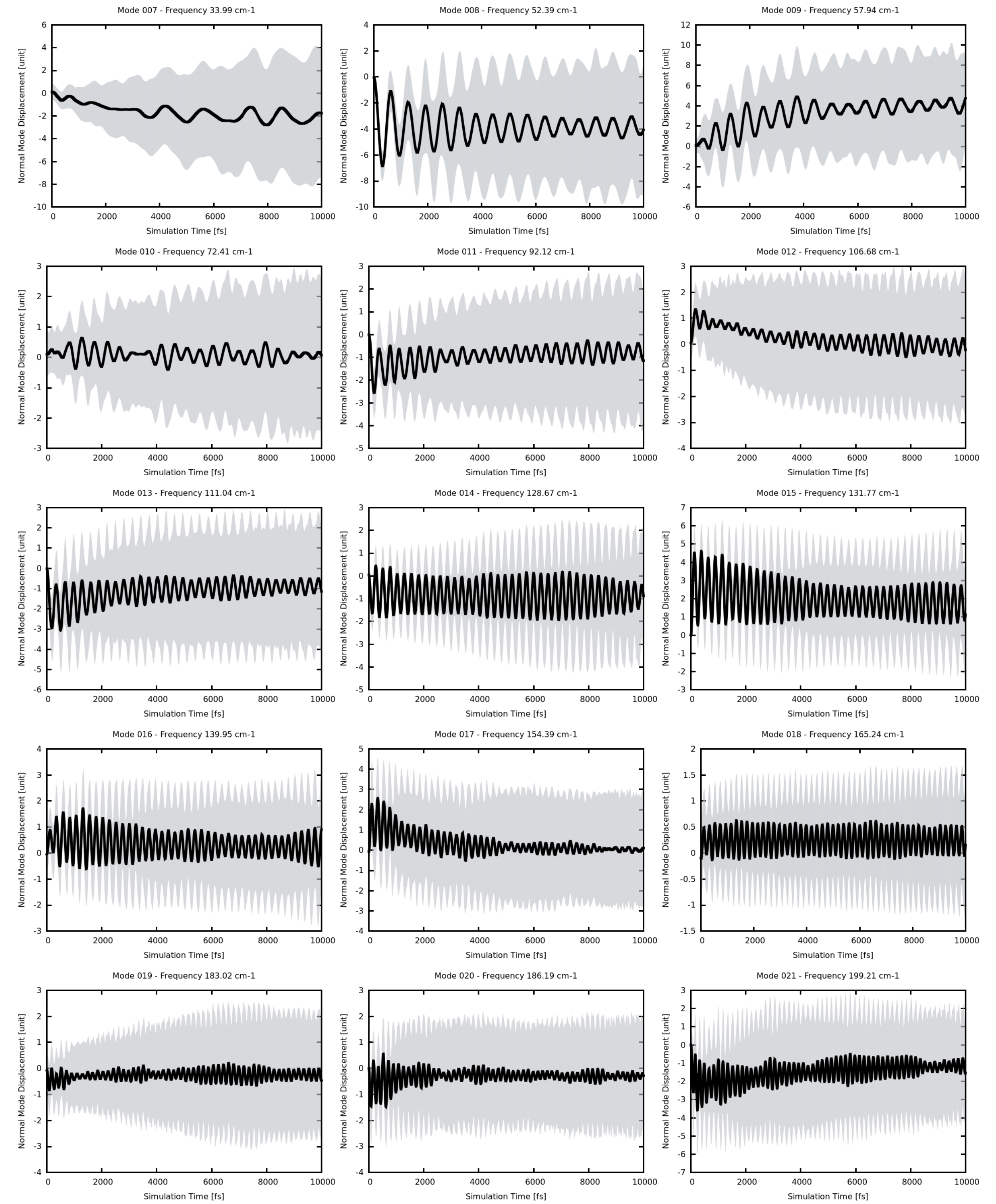

Figure S11 continued

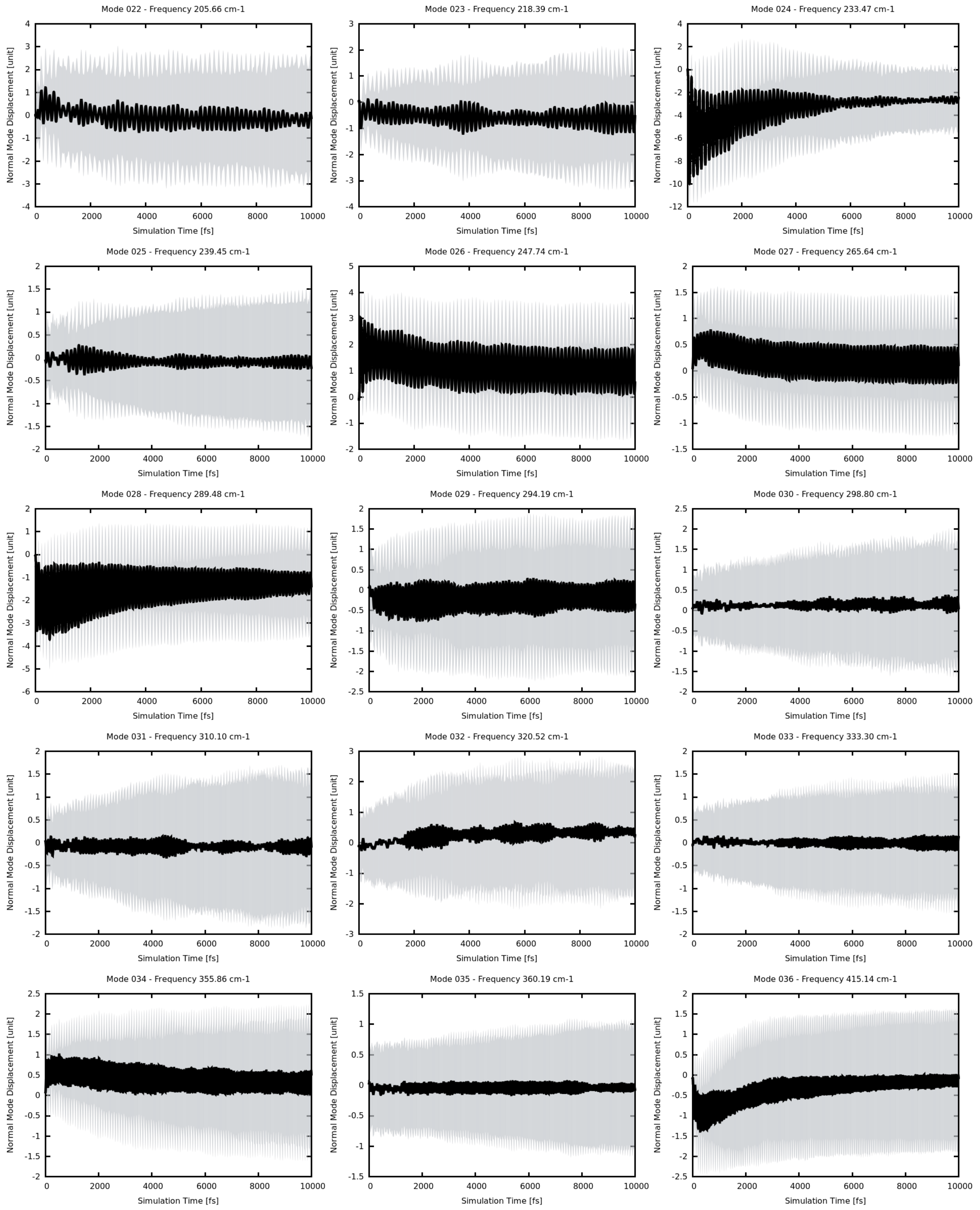

Figure S11 continued

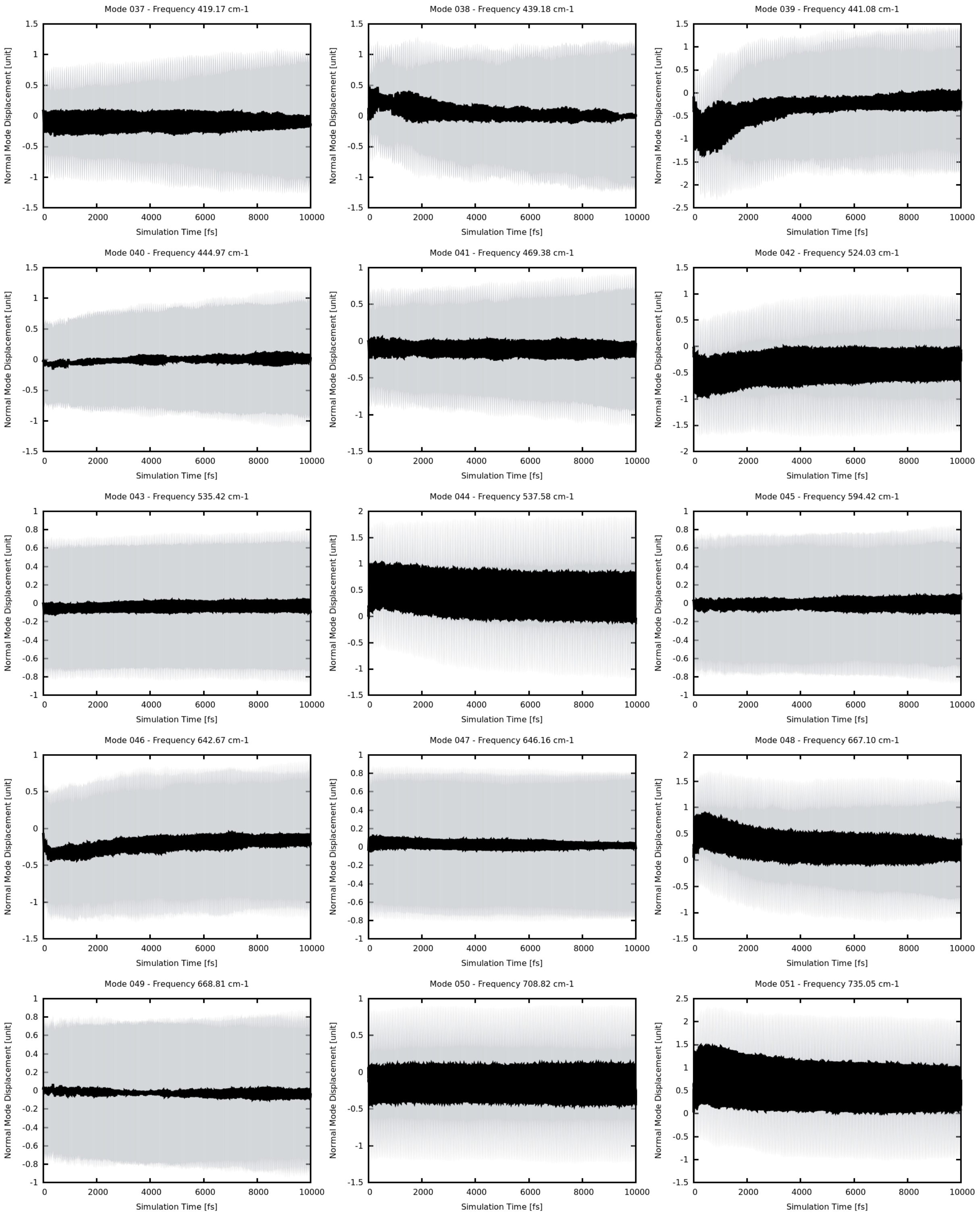

Figure S11 continued

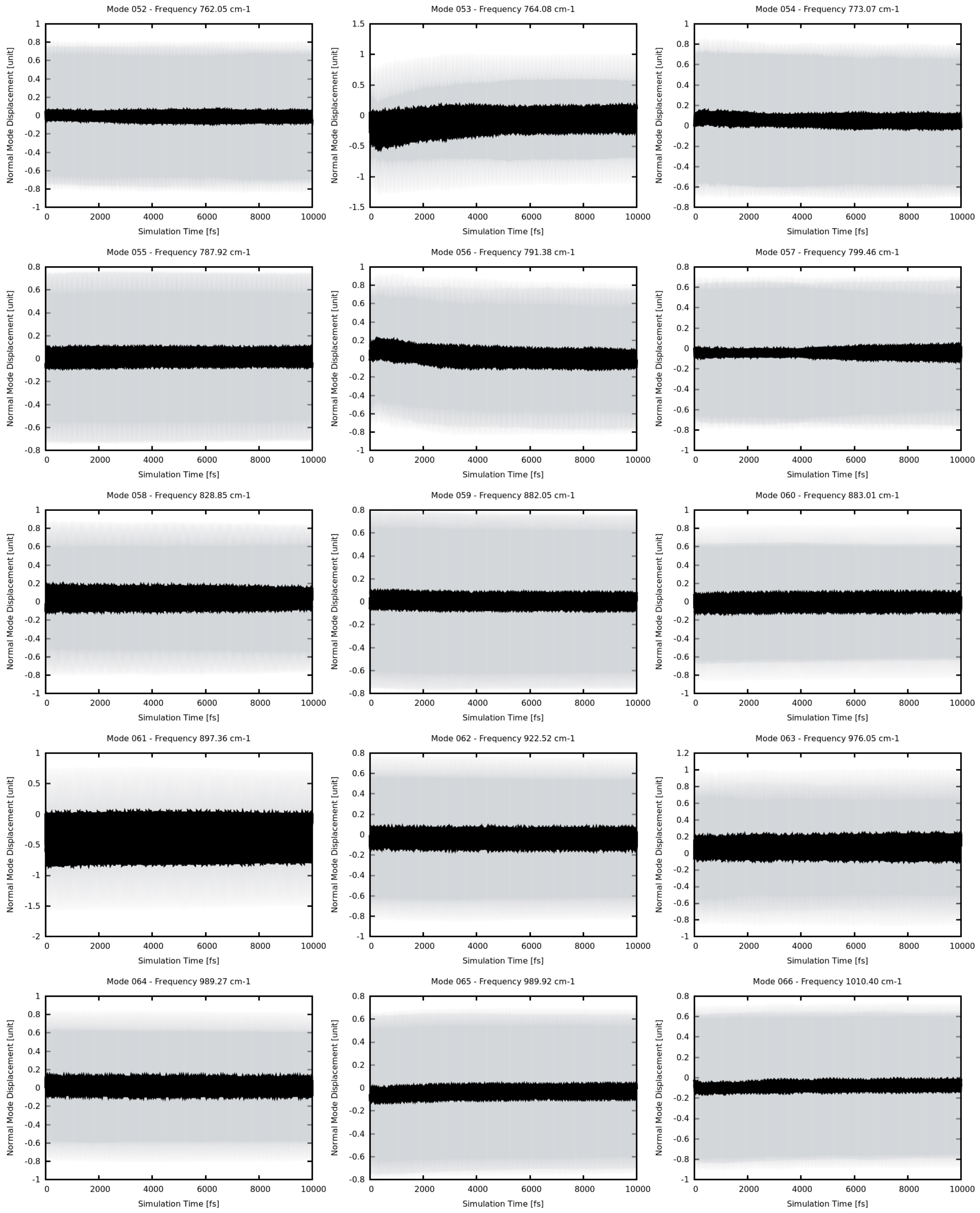

Figure S11 continued

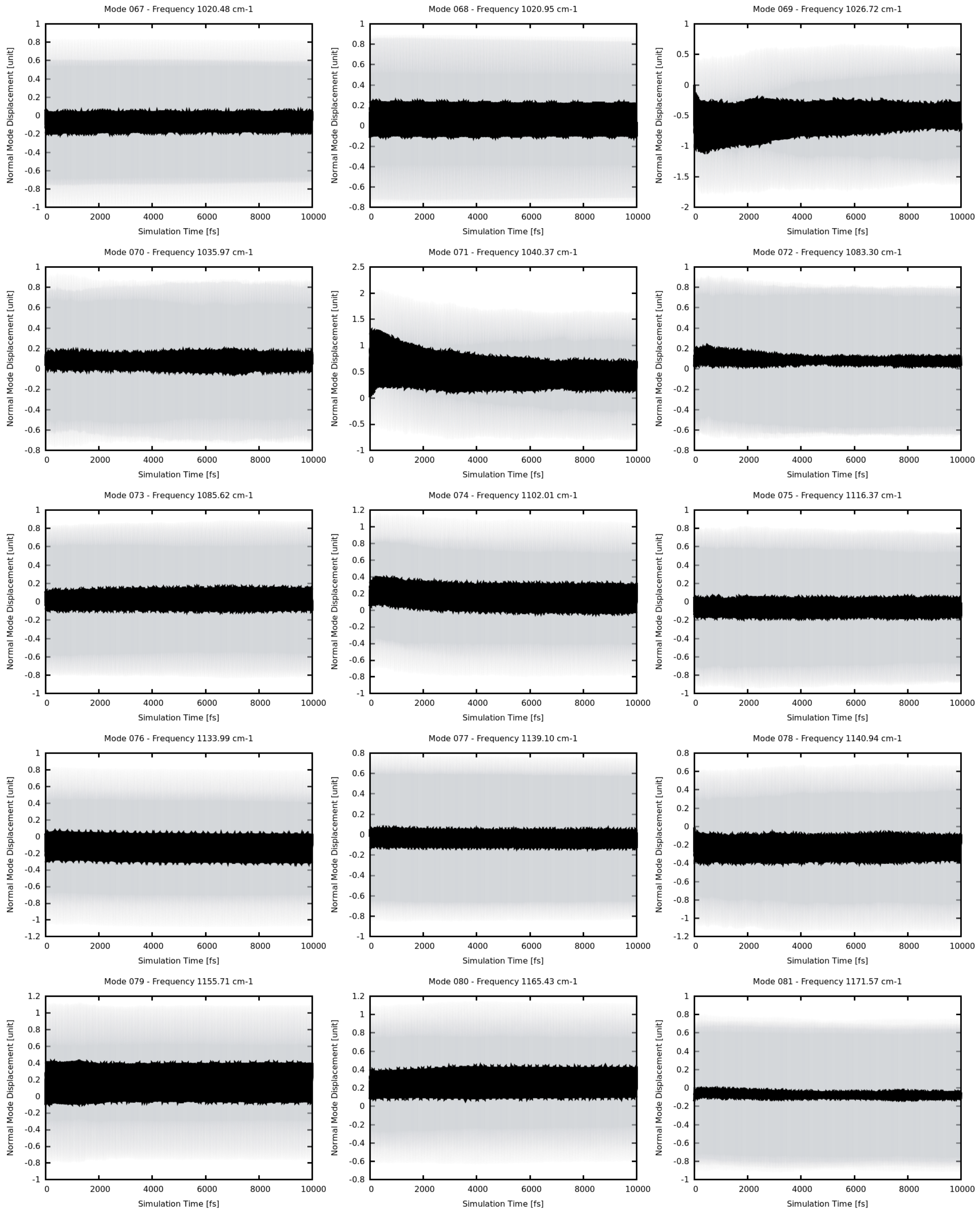

Figure S11 continued

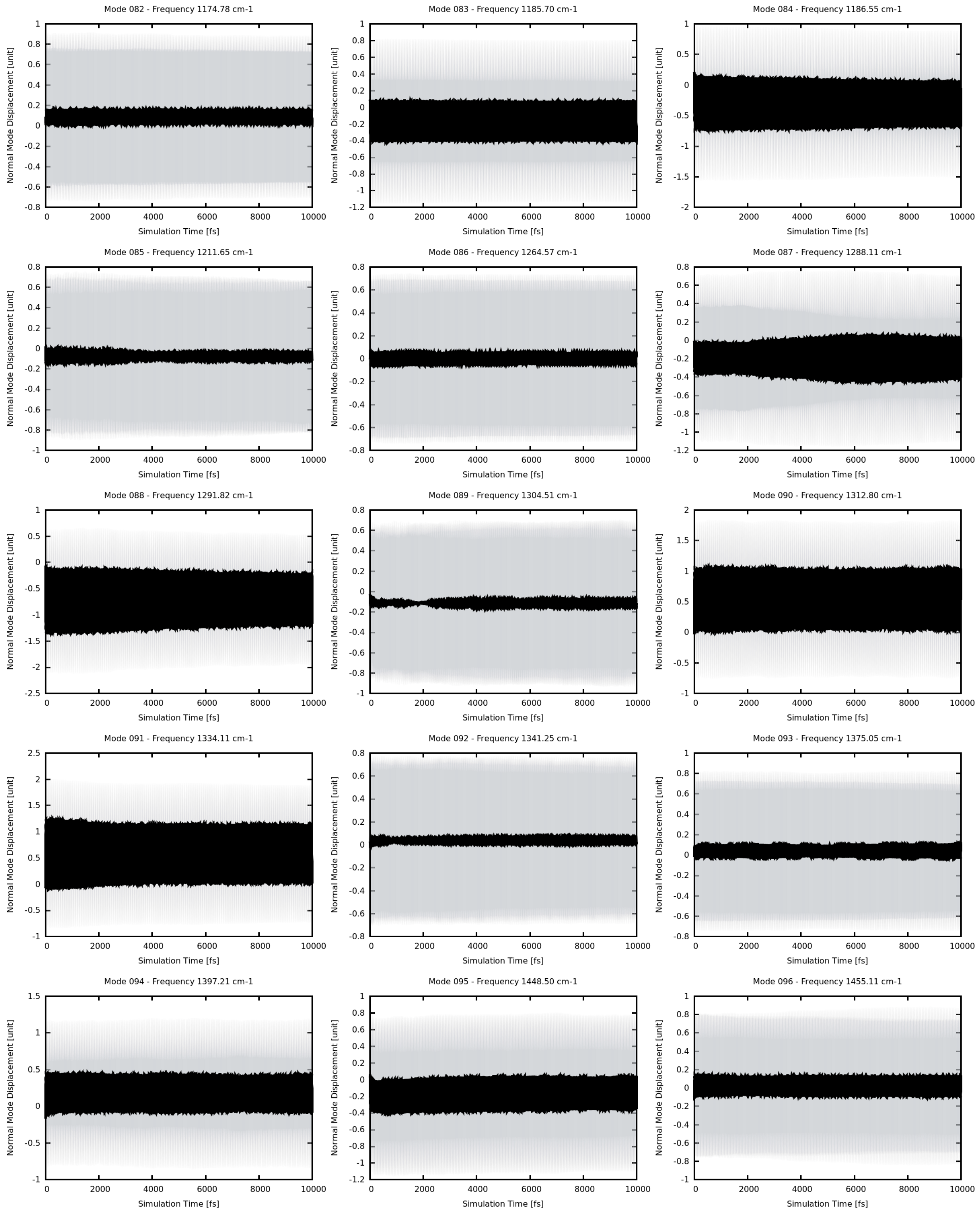

Figure S11 continued

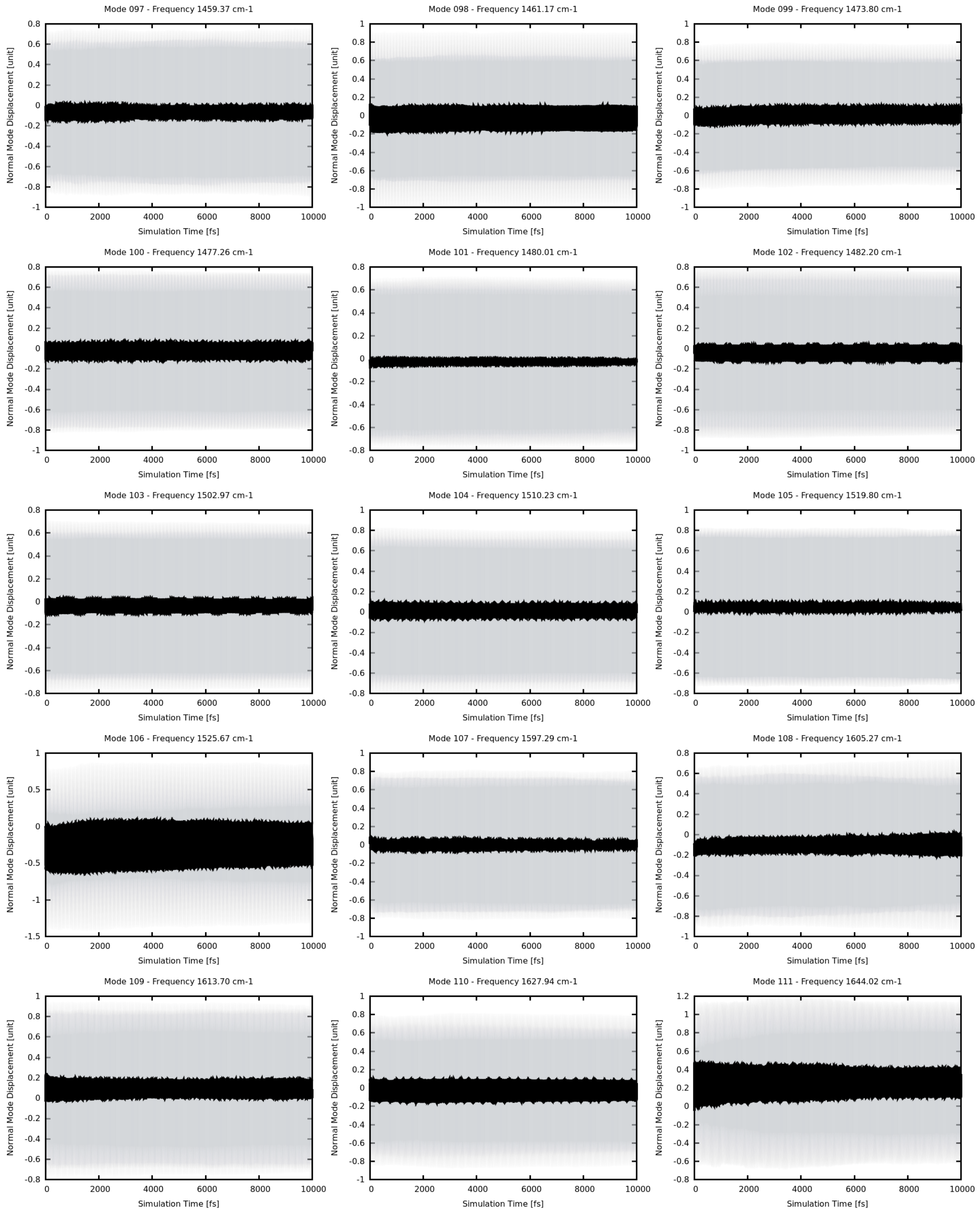

Figure S11 continued

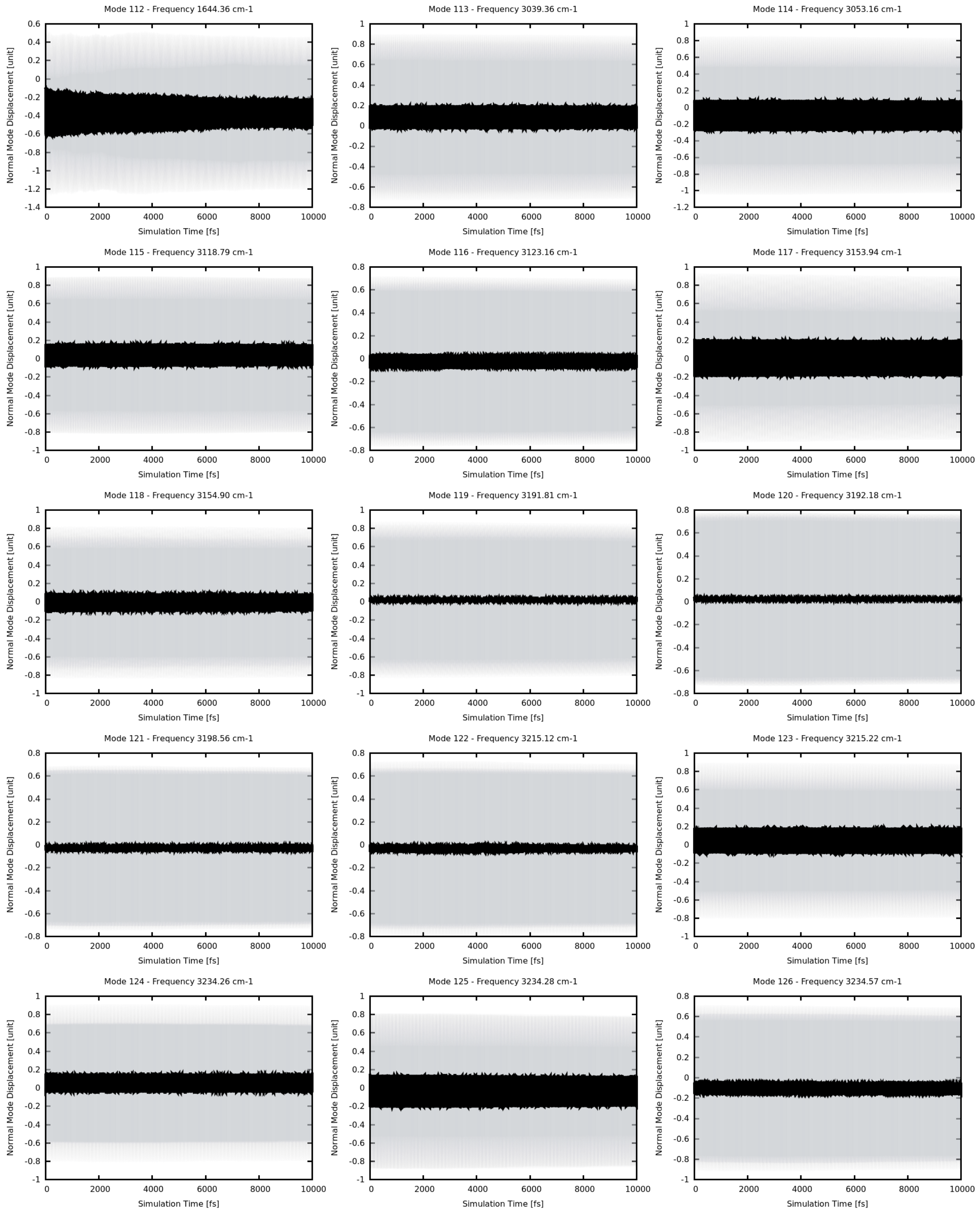

Figure S11 continued

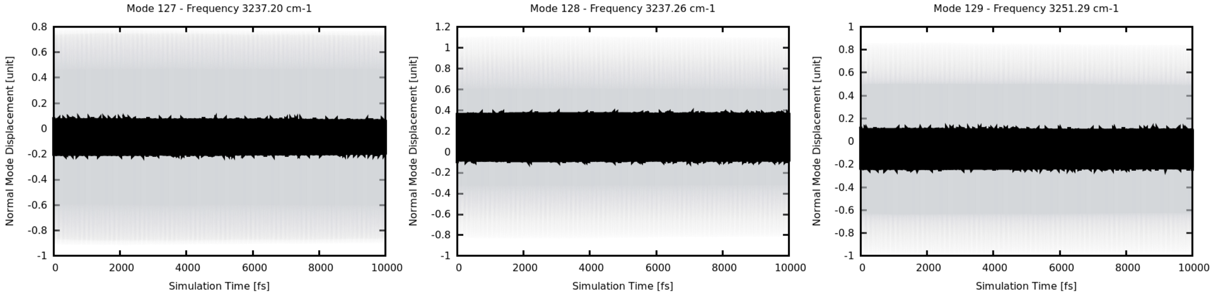

S4.7 Internal Coordinate Analysis

In the main paper, we show the time evolution of selected internal coordinates for the full simulation time of 0-10 ps in Figure 5. Here, we zoom into the 0-200 fs time window for the same coordinates in Figure S12. We can see that the averages (blue lines) of all V-N and V-Cl distances as well as the angle between the atoms $N_3 - V - N_4$ start at values are very close to the reference value of the FC geometry (red line). For the angles between the atoms $Cl_{41} - V - N_2$ and $Cl_{42} - V - Cl_{43}$, there is a slight difference between the initial values of the averages of the trajectories (176 and 177°) and that of the FC reference geometry (180 and 180°), that is an effect of the vibrational sampling from the Wigner distribution.

In addition, we have analyzed the time evolution of selected internal coordinates of trajectories exclusively in singlet states, see Figure S13. This was done by selecting trajectories where the active state possesses a spin expectation value of $\langle S^2 \rangle > 0.5$. We can see that the time evolution of the trajectories in the singlet states is similar to that of the whole ensemble –which is representative for the trajectories in the triplet states, as these account for at least 90 % of all trajectories at all simulation times (see Figure 4 in the main paper.) However, notable differences can be seen for the first 5 ps, e.g., in the magnitude of the V−N distances, that are smaller for the singlet trajectories than for the whole ensemble. This can be explained considering that most of the ensemble traverses the ^{3}MC$_4$ triplet state, which

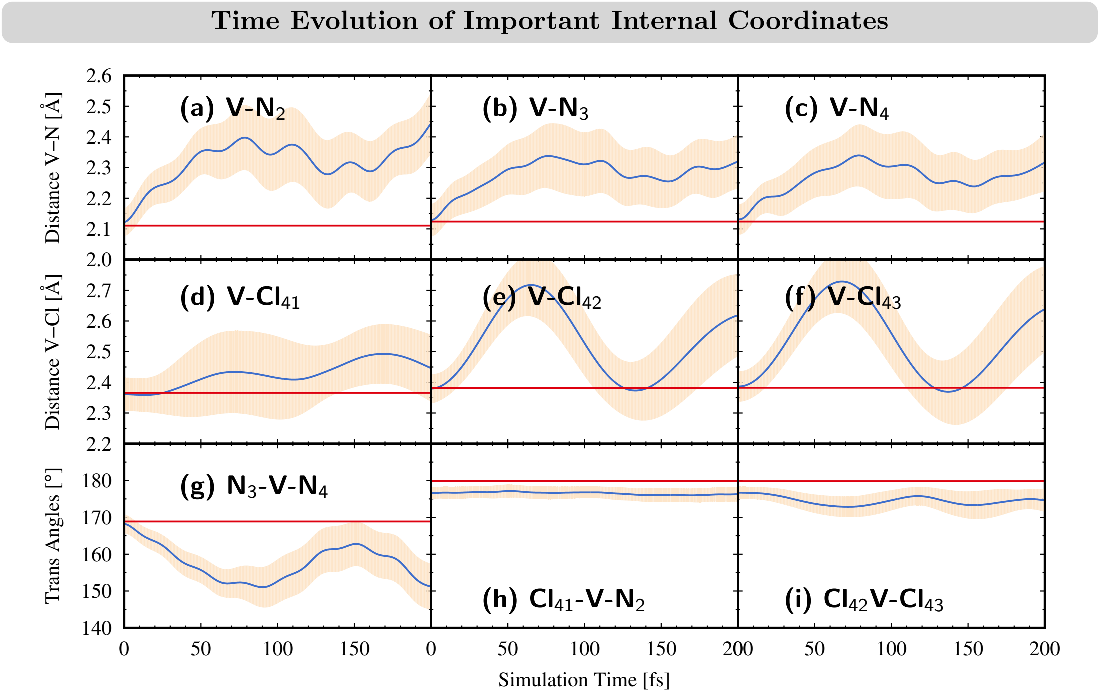

Figure S12: Time evolution of important internal coordinates for simulation times 0-200 fs. (a-c) V-N distances. (d-f) V-Cl distances. (g-i) Trans angles in the complex. Blue line follow the average value of the 200 trajectories, while the filled orange area depicts the standard deviation. Red line shows the value at the FC geometry.

possesses a $(e_g)^2$ configuration, i.e., with two electrons in the antibonding e_g orbitals

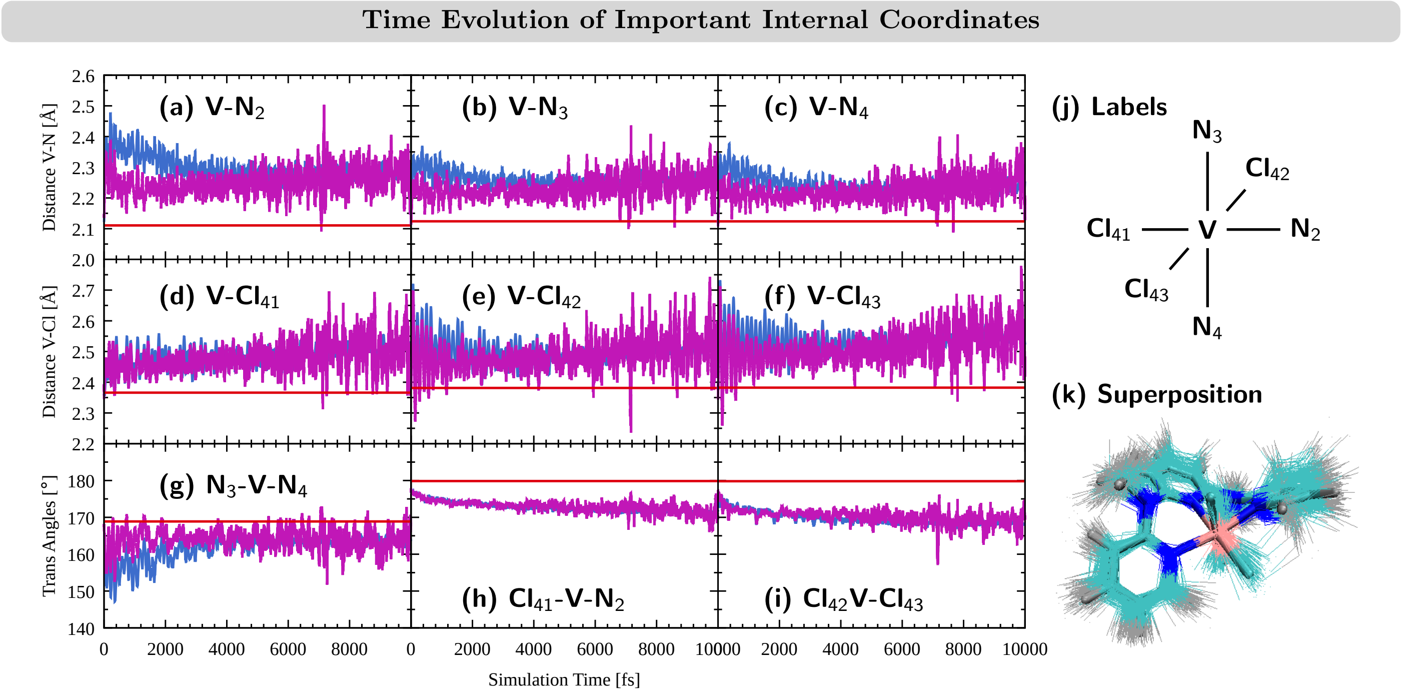

Figure S13: Time evolution of important internal coordinates for simulation times 0-10 ps. (a-c) V-N distances. (d-f) V-Cl distances. (g-i) Trans angles in the complex. Blue line follow the average value of the 2000 trajectories, while purple lines follow the average of trajectories in singlet states. Red line shows the value at the FC geometry.

S4.8 CASSCF vs. LVC Potential Energy Curves

In this work, we have used LVC potentials parameterized at the CASSCF level of theory. The parameterization was performed at the Franck-Condon geometry of the T_0 ground state. It is now interesting to investigate how well the LVC potentials can approximate the CASSCF potentials at geometries displaced from the FC geometry. To test this, we have computed one-dimensional potential energy curves of the triplet states along some of the most active normal modes –as identified in Section S4.6. The calculations have been performed at the CASSCF(10,13) level of theory and using the parameterized LVC models. The resulting PES for the normal modes 7, 9, 12, 32, 36, and 39 are shown in Figure S14 alongside the average displacement (plus standard deviation) of the trajectories during the dynamics (repeated from Figure S11). The CASSCF curves are thereby given as solid lines, while the LVC curves are presented as dashed lines. As can be seen, for modes 7, 9, 12, 36, and 39, the LVC potentials approximate the CASSCF potentials also for large normal mode displacements well for all 16 triplet states. For normal mode 32, the LVC potential can approximate the CASSCF potential only well for negative displacements. This mode can be mainly characterized by an antisymmetric $Cl_{41}-V-N_2$ stretching vibration (see also the vcl3ddpd.molden file given as additional supporting information), whereby a negative displacement increases the V_{N2} distance and decreases the C−41−V distance. For positive displacements (shortened V_N distance, prolonged $Cl_{41}-V$ distance), the LVC curves increase too slowly for displacements larger than 2 arb. units. Thus, one may suspect, that in the dynamics using the LVC potentials, the trajectories could reach artificially large displacements along this normal mode coordinate. However, as can be seen next to the curves of mode 32, the average normal mode displacement of the trajectories throughout the dynamics lies between -0.1 and 0.5 with the standard deviation typically accounting for an interval of ± 1.5. Thus, the fraction or trajectories of the ensemble that reaches the regions of displacements $> +2$ where the LVC potentials deviate considerably from the CASSCF potentials is very small. Importantly, such large displacements are only reached at later times in the dynamics –after ca. 3 ps –and,

thus, do not influence on the ISC processes.

Figure S14: Left-hand side: CASSCF(10,13) (solid line) vs. LVC (dashed lines) potential energy curves for the triplet states long selected normal mode displacements. Right-hand side: Time evolution of normal-mode displacements of the ensemble of trajectories repeated from Figure S11

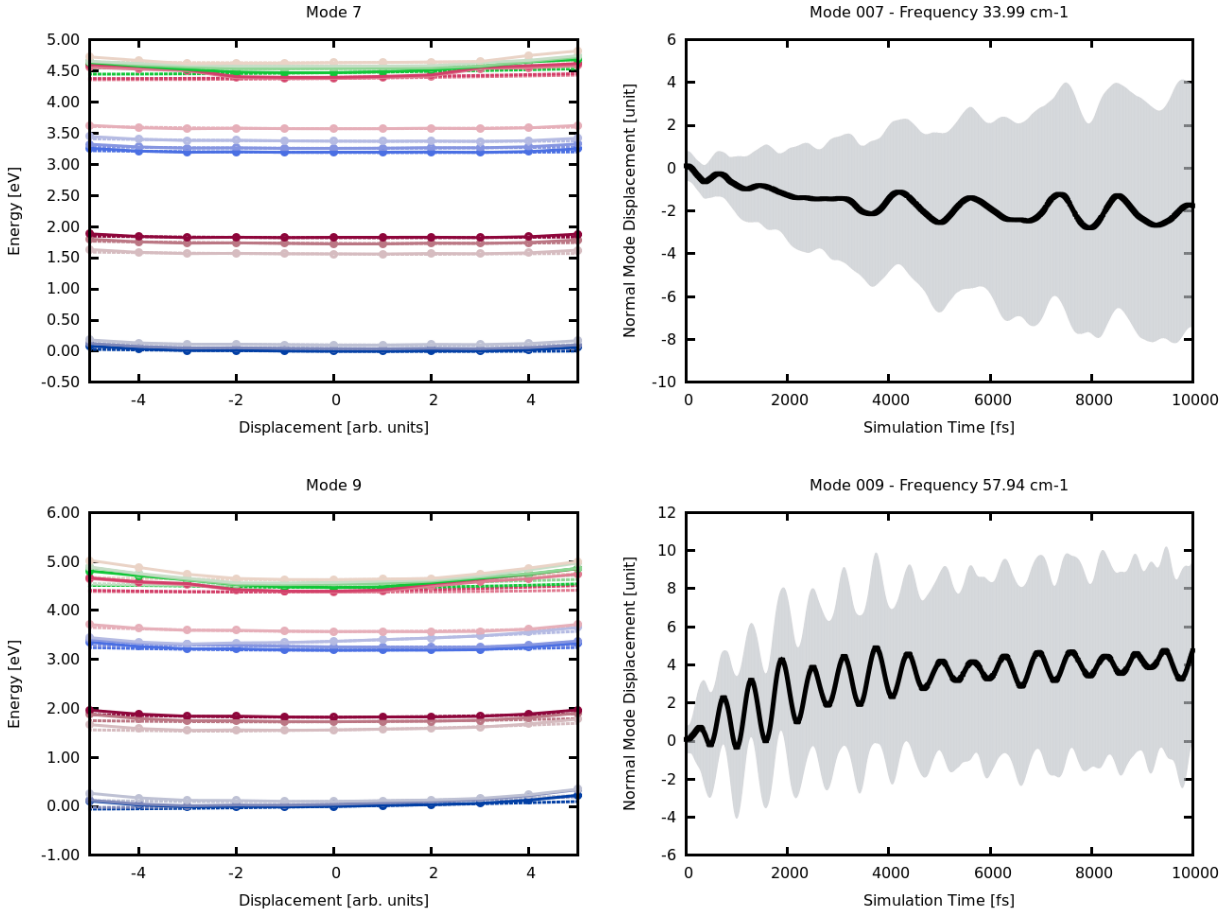

Figure S14 continued

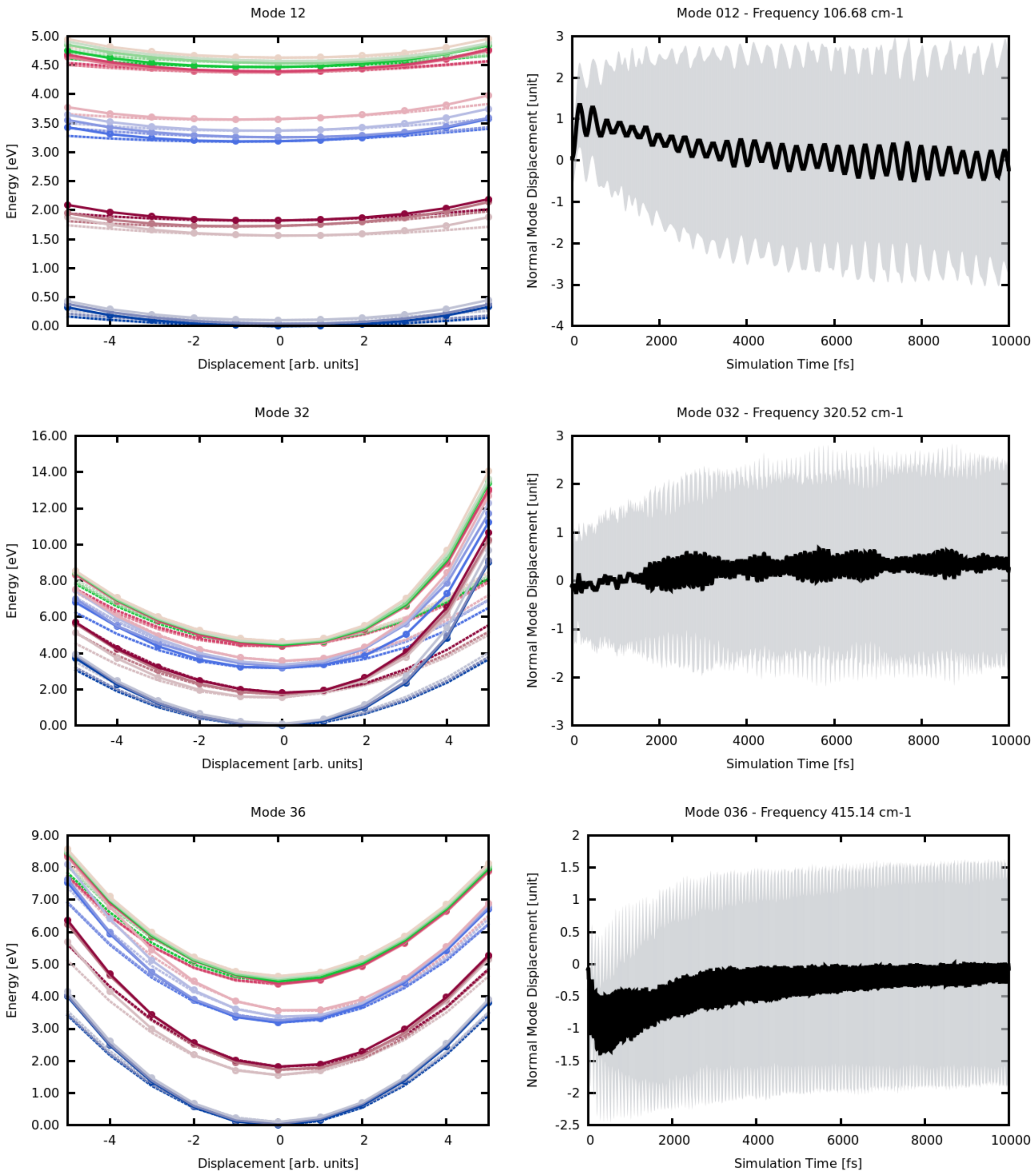

Figure S14 continued

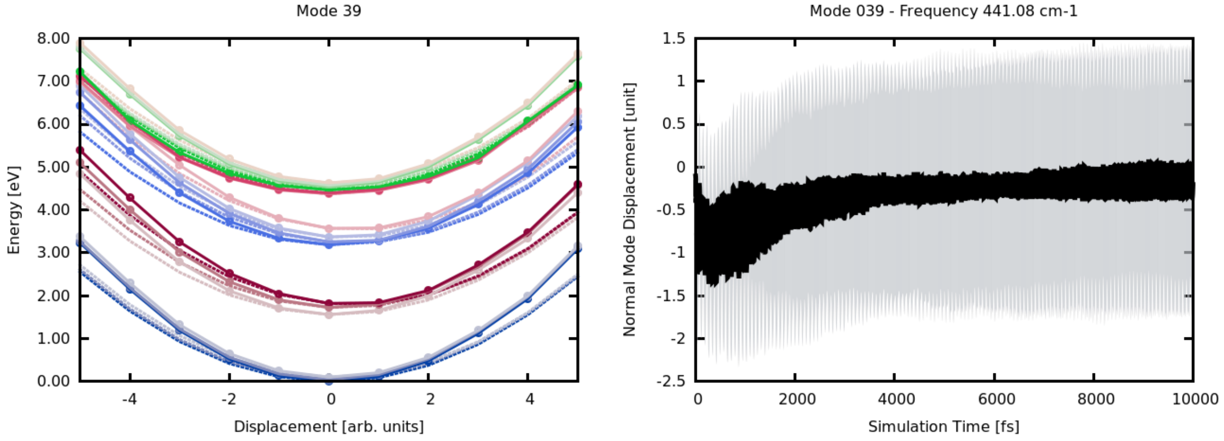

References

(1) Plasser, F. TheoDORE: A toolbox for a detailed and automated analysis of electronic state computations. *J. Chem. Phys.* **2020**, *152*, 084108.

(2) Galván, I. F.; Vacher, M.; Alavi, A.; Angeli, C.; Aquilante, F.; Autschbach, J.; Bao, J. J.; Bokarev, S. I.; Bogdanov, N. A.; Carlson, R. K.; Chibotaru, L. F.; Creutzberg, J.; Dattani, N.; Delcey, M. G.; Dong, S. S.; Dreuw, A.; Freitag, L.; Frutos, L. M.; Gagliardi, L.; Gendron, F.; Giussani, A.; González, L.; Grell, G.; Guo, M.; Hoyer, C. E.; Johansson, M.; Keller, S.; Knecht, S.; Kovačević, G.; Källman, E.; Manni, G. L.; Lundberg, M.; Ma, Y.; Mai, S.; Malhado, J. P.; Malmqvist, P. Å.; Marquetand, P.; Mewes, S. A.; Norell, J.; Olivucci, M.; Oppel, M.; Phung, Q. M.; Pierloot, K.; Plasser, F.; Reiher, M.; Sand, A. M.; Schapiro, I.; Sharma, P.; Stein, C. J.; Sørensen, L. K.; Truhlar, D. G.; Ugandi, M.; Ungur, L.; Valentini, A.; Vancoillie, S.; Veryazov, V.; Weser, O.; Wesołowski, T. A.; Widmark, P.-O.; Wouters, S.; Zech, A.; Zobel, J. P.; Lindh, R. OpenMolcas: From Source Code to Insight. *J. Chem. Theory Comp.* **2019**, *15*, 5925–5964.

(3) Mai, S.; González, L. Molecular Photochemistry: Recent Developments in Theory. *Angew. Chem. Int. Ed.* **2020**, *59*, 16832–16846.

(4) Ghigo, G.; Roos, B. O.; Malmqvist, P.-Å. A modified definition of the zeroth-order Hamiltonian in multiconfigurational perturbation theory CASPT2. *Chem. Phys. Lett.* **2004**, *396*, 142–149.

(5) Zobel, J. P.; Nogueira, J. J.; González, L. The IPEA Dilemma in CASPT2. *Chem. Sci.* **2017**, *8*, 1482–1499.
